# Correction: Physicians’ narratives of communication with patients and their relatives in different phases of the palliative pathway

**DOI:** 10.1136/bmjopen-2022-065681corr1

**Published:** 2023-12-01

Landstad BJ, Kvangarsnes M. Physicians’ narratives of communication with patients and their relatives in different phases of the palliative pathway. *BMJ Open* 2023;13:e065681. doi: 10.1136/bmjopen-2022-065681.

This article was previously published with an error.

The authors of this article wish to draw attention to the updated author list, which now includes an additional author. As such, the author list and the Contributor statement has been updated accordingly.

