# Supplementary material for: Physicians’ narratives of communication with patients and their relatives in different phases of the palliative pathway
Source: BMJ Open. 2023 Jun 16;13(6):e065681. doi: 10.1136/bmjopen-2022-065681 (PMC10277073; doi:10.1136/bmjopen-2022-065681)
Supplement: Author's manuscript [file bmjopen-2022-065681.draft_revisions.pdf]

BMJ Open

BMJ Open is committed to open peer review. As part of this commitment we make the peer review history of every article we publish publicly available.

When an article is published we post the peer reviewers' comments and the authors' responses online. We also post the versions of the paper that were used during peer review. These are the versions that the peer review comments apply to.

The versions of the paper that follow are the versions that were submitted during the peer review process. They are not the versions of record or the final published versions. They should not be cited or distributed as the published version of this manuscript.

BMJ Open is an open access journal and the full, final, typeset and author-corrected version of record of the manuscript is available on our site with no access controls, subscription charges or pay-per-view fees (http://bmjopen.bmj.com).

If you have any questions on BMJ Open's open peer review process please email info.bmjopen@bmj.com

BMJ Open

Physicians’ narratives of communication with patients and their relatives in palliative care

Journal:	*BMJ Open*
Manuscript ID	bmjopen-2022-065681
Article Type:	Original research
Date Submitted by the Author:	17-Jun-2022

Complete List of Authors:	Landstad, Bodil; Mid Sweden University Faculty of Human Science, Health Sciences; Östersund Hospital, Unit of Research, Education and Development<br>Kvangarsnes, Marit; Norwegian University of Science and Technology; Møre og Romsdal Hospital Trust

Keywords:	Quality in health care < HEALTH SERVICES ADMINISTRATION & MANAGEMENT, Adult palliative care < PALLIATIVE CARE, QUALITATIVE RESEARCH

SCHOLARONE™
Manuscripts

I, the Submitting Author has the right to grant and does grant on behalf of all authors of the Work (as defined in the below author licence), an exclusive licence and/or a non-exclusive licence for contributions from authors who are: i) UK Crown employees; ii) where BMJ has agreed a CC-BY licence shall apply, and/or iii) in accordance with the terms applicable for US Federal Government officers or employees acting as part of their official duties; on a worldwide, perpetual, irrevocable, royalty-free basis to BMJ Publishing Group Ltd ("BMJ") its licensees and where the relevant Journal is co-owned by BMJ to the co-owners of the Journal, to publish the Work in this journal and any other BMJ products and to exploit all rights, as set out in our licence.

The Submitting Author accepts and understands that any supply made under these terms is made by BMJ to the Submitting Author unless you are acting as an employee on behalf of your employer or a postgraduate student of an affiliated institution which is paying any applicable article publishing charge ("APC") for Open Access articles. Where the Submitting Author wishes to make the Work available on an Open Access basis (and intends to pay the relevant APC), the terms of reuse of such Open Access shall be governed by a Creative Commons licence – details of these licences and which Creative Commons licence will apply to this Work are set out in our licence referred to above.

Other than as permitted in any relevant BMJ Author's Self Archiving Policies, I confirm this Work has not been accepted for publication elsewhere, is not being considered for publication elsewhere and does not duplicate material already published. I confirm all authors consent to publication of this Work and authorise the granting of this licence.

For peer review only

Title of the article

Physicians' narratives of communication with patients and their relatives in palliative care

Authors

1. LANDSTAD Bodil J.*
    a. Department of Health Sciences, Mid Sweden University, Sweden
    b. Unit of Research, Education and Development, Östersund Hospital, Östersund, Sweden

    Address: Department of Health Sciences, Mid Sweden University, Campus Östersund, 831 25 Östersund, Sweden

    E-mail: bodil.landstad@miun.se

    Phone: +46 730912884

    Fax: -

2. KVANGARSNES Marit
    a. Department of Health Sciences, Norwegian University of Science and Technology, Ålesund, Norway
    b. Møre og Romsdal Hospital Trust, Ålesund, Norway

*Corresponding author

Keywords

Physicians, qualitative research, communication, palliative care, clinical pathway

Word count

3 855(excluding title page, abstract, references)

Abstract

Objectives: To explore physicians communicating with patients and their relatives in palliative care.

Methods: Purposeful sampling was employed to recruit a total of 13 oncologists and general practitioners who engaged in palliative care in specialist's health care and primary care. A qualitative

For peer review only - http://bmjopen.bmj.com/site/about/guidelines.xhtml

study with a narrative approach was conducted. Interviews with physicians working in primary and specialist health care were employed via Skype Business in Spring 2020. We used an interview guide with open-ended questions. Each interview lasted between 35 and 60 minutes.

Results: The physicians told that the communication with patients and their relatives was contextual, and changed depending on the phase. In the early phase, physicians expressed that patients and their relatives experienced emotional shock. Transitioning from the curative to palliative phase was difficult, thus emphasising the need for trust during communication. In the middle phase, they revealed that communication about the death process became essential. During the terminal phase, physicians employed a compassionate approach, as bereaved individuals needed to process guilt and grief. It was important for the physicians to communicate information about the palliative pathway while providing the relatives knowledge that facilitates decision making.

Conclusions: Focusing on the physician's perspective, the narrative approach produced insight into communication with patients and their relatives during different phases of palliative care. The findings should help physicians improve the quality of communication with patients and their relatives over these vulnerable pathways. The findings have practical implications in training contexts.

Strengths and limitations of this study

- The interviews provided rich and complex data
- The theoretical framework revealed theoretical perspectives for the analysis
- The researchers had different professional backgrounds
- The study was solely conducted in the Norwegian context

What is known on this topic

For peer review only - http://bmjopen.bmj.com/site/about/guidelines.xhtml

For peer review only

- To provide quality palliative care, it is highly important for physicians to communicate with patients and their relatives, but this entails an asymmetrical relationship that is linked to the physician's professional role.
- It is difficult to communicate about issues pertaining to the transition from curative to palliative care.

What this study adds

- Physicians conveyed that communication was important, especially regarding the responsibility to decide on courses of treatment based on their expertise.
- The results expand on current knowledge related to what patients and their relatives need to know about different phases of the pathway.

How this study might affect research, practice or policy

- Additional studies should continue to investigate the issue with cultural dimension of communication in palliative care. It is also of interest to study communication in team working between health professionals in palliative care.
- The digital revolution opens up new ways of communicating and it would be of great interests to explore possibilities of interacting in palliative care.

For peer review only

INTRODUCTION

Health care personnel's communication with patients and their relatives in palliative care is crucial.[1] The physicians are responsible for the medical treatment and person cantered care. Although palliative care is seen as an ethical responsibility, global estimates show that only 14% of patients in need actually receive it.[1] In previous research, the Lancet Oncology Commission suggested the need to integrate the tumour-directed and patient-centred approaches, thus focusing on the experiences of individuals suffering from advanced disease and who may require palliative care.[2] In this study, we

For peer review only - http://bmjopen.bmj.com/site/about/guidelines.xhtml

used the definition for palliative care provided by the World Health Organization (WHO), which states that it is an approach that improves the quality of life of patients and their families who are facing problems associated with life-threatening illness. It prevents and relieves suffering through the early identification, correct assessment and treatment of pain and other problems, whether physical, psychosocial or spiritual'.[1] Palliative care is thus an interdisciplinary approach in which physicians play important roles. As such, this study focused on communication with patients and their relatives by soliciting narratives of communication from the physician's perspective, as this is a highly important component for ensuring quality care.[3] In this context, communication refers to the interpersonal activity of expressing ideas and feelings or providing information; as derived from Latin, communication also refers to 'something you have in common'.[4]

Time and space create the contextual conditions for communication,[5] in which case the physical framework also serves as an important basis. This investigation focused on the type of communication that occurs between physicians and patients/relatives in hospitals, home environments, or municipal health service institutions. It is an asymmetrical form of communication with a clear purpose. Here, the patient is in a vulnerable position due to both their dependence on the physician's competence and access to treatment.[5] As a result of this vulnerability, any such communication is linked to the physician's professional role and position within the specific system, which makes symmetrical communication undesirable and unprofessional. The relative power held by the physician in this role also entails the risk that patients and their relatives will experience dependency and shame.[6]

A Swedish study on experiences with end-of-life communication showed that some physicians were uncertain about prognoses, which created a communication barrier and might hindered treatment decisions.[7] The complex nature of the transition from curative to palliative treatment may also instil uncertainty and diminish the potential for good dialogues.

For peer review only - http://bmjopen.bmj.com/site/about/guidelines.xhtml

Sympathy, empathy, and compassion are important for patients at the end stage of life, but research has shown that patients place the highest value on compassion.[8] A systematic review of 31 studies aimed at communication between healthcare professionals and the relatives of patients approaching the end-of-life stage identified seven relevant themes, including highlighting deterioration, involvement in decision-making, post-decision interactional work, tailoring, honesty and clarity, specific techniques for information delivery, and roles of different healthcare professionals in communication.[9]

A study in New Zealand found that experiences of emotional shock and grief after learning about a life-limiting illness made it very difficult to hear and process health information.[10] As such, health professionals may avoid having conversations about moving from active treatment to palliative care, which may create additional distress and confusion when making choices after prognosis. The same study showed that a lack of cultural health literacy among health professionals led to late access or the avoidance of palliative care among indigenous peoples.[10] In short, patient-centred care and compassion is paramount for patients and relatives during communication in palliative care.[11, 12, 13, 14] The objective of the study is to explore physicians communicating with patients and their relatives in palliative care. There is a lack of knowledge about how to communicate in different phases of palliative care from the physician's perspective. We posed the following question: How do physicians experience communication with patients and relatives in the context of palliative care?

METHODS

We employed a narrative approach and subsequent analysis, which entails different theoretical directions that capture personal and human dimensions of experience over time by considering the relationship between individual experiences and cultural contexts.[15] Here, the narrative approach concerned how physicians perceived communication in the beginning, middle, and terminal phases

For peer review only - http://bmjopen.bmj.com/site/about/guidelines.xhtml

of palliative care based on interview data.[16, 17] The study compiled with the consolidated criteria for reporting qualitative research (COREQ).[18]

Data and study population

The physicians were recruited by purposive sampling aimed at capturing the complexity of the palliative pathway.[16, 19] We chose physicians with experience in the beginning, middle, and terminal phases.

Using a contact person, we recruited a total of 13 physicians working in palliative care settings. To ensure diversity and robustness, we recruited a mixture of males and females of various age groups, experience levels, and medical backgrounds. Table 1 shows the demographic data.

Table 1. Demographic data on the study participants.

	**Participants** (N=13)
**Gender**	
Men	10
Women	3
**Age**	
41-50	7
51-60	4
61-70	2
**Place of assignment***	
Hospital	7
Primary care	7
Private clinic	1
**Education****	

For peer review only - http://bmjopen.bmj.com/site/about/guidelines.xhtml

General practitioner	5
Oncologist	5
Physician	5
**Years of experience**	
10-15	2
16-20	5
21-25	-
26-30	4
>30	2

*Some physicians reported more than one work site.

**Some physicians worked as both oncologists and general practitioners.

Individual interviews were conducted via Skype Business between April and May 2020. For this purpose, we developed an interview guide (See Table 2) with open-ended questions based on the study aim and previous research.[20] This facilitated the solicitation of personal experiences related to communication over the palliative pathway.[21] More specifically, the questions were related to how physicians perceived communication during different phases of the palliative pathway. Each interview lasted between 35 and 60 minutes. The interviewer was a researcher and cancer nurse, trained to conduct interviews.[18] Saturation was gained upon ceasing to result in new information related to the different phases.[16] After 13 interviews the data were rich and thick[20] related to the different phases of the palliative pathway. We considered the data to be saturated.

Table 2. Interview guide.

	**Question**
1	How do you expiring being a physician in palliative care?

For peer review only - http://bmjopen.bmj.com/site/about/guidelines.xhtml

2	What is important in communication with patients and relatives in the first phase of the illness?
3	Can you tell about your communication in the middle phase of the pathway?
4	How do you cooperate with patients and relatives?
5	What is of importance in the terminal phase?
6	Is there anything else you want to share with us?

Patient and public involvement

None.

Data analysis

All interviews were audio-recorded and transcribed verbatim. We then read these transcripts to gain a holistic impression of the data.[20] The result section contains stories from 13 physicians that forms one narrative. The 13 physicians had rich and thick experiences from all phases of the palliative pathway. A narrative does not represent “the truth”. In narrative research the researchers are co-creators in shaping the narrative. A narrative is formed by the way we make meaning of our experiences. This means how we understand our own or others’ actions and the way we organise events. How we connect and perceive the consequences of actions over time is of importance for the shaping of the narrative. Meaning making of our experiences can be linked to the past, the present, and the future.[20]

During a subsequent content analysis, we searched for a plot based on patterns in the narratives. Specifically, this plot described communication during the early, middle, and terminal phases of the palliative pathway.[16, 17] We thus derived and analysed three main communication themes, which are

For peer review only - http://bmjopen.bmj.com/site/about/guidelines.xhtml

described later in the Results section. In Table 3 we present an example of analysis process for one theme.

Table 3. Example of analysis process.

**Quotation**	**Subtheme**	**Main theme**
We should take the challenge of talking to patients and relatives together, because they need to hear what the other person is saying - they need to hear together what we are saying.	Common understanding	Communication about the dying process
If we're going to come in and directly inform about prognosis and ask what they want, how they want to die, then I think it's scary - it's too brutal.	Compassion	
We have to accept [situations in which] we do not achieve our treatment goals or a 'good death', which is a value for us. Ain't no cure for death.	Respect for the choices of patients and their relatives	

For peer review only - http://bmjopen.bmj.com/site/about/guidelines.xhtml

Ethical consideration

All participants were informed about the study purpose and procedures in oral and written formats. They were told that all participation was voluntary and that they could withdraw at any time. Further, they were informed that any obtained data would be handled confidentially. The physicians provided informed consent and the consent was written.

RESULTS

Oncologists and general practitioners (GPs) who worked in hospitals or primary care settings provided information about how they supported patients and their relatives during different phases of the palliative care process. We therefore derived the following three themes related to the early, middle, and terminal stages, respectively: 1) patients and their relatives in crises, 2) communication about the death process, and 3) processing relatives' guilt and grief.

Patients and their relatives in crises

The narratives revealed that the transition from curative to palliative care was difficult. In addition to disease-related ailments, patients and their relatives underwent an internalised process of acceptance. This often entailed feelings of shock, especially for patients who gained insight into their limited lifespans. This was also often the beginning of a mental process leading to cognition and acceptance of death. Here, patients had to avail of resources, networks, and coping strategies they acquired earlier in their lives. Time was marked by loss, and the patients learned they should not expect improvement.

Physicians expressed they wanted to talk with patients and their relatives together. Here, the task was to help patients and their relatives develop a common understanding of the specific situation in palliative care. One participant said the following:

For peer review only - http://bmjopen.bmj.com/site/about/guidelines.xhtml

‘We should take the challenge of talking to patients and relatives together, because they need to hear what the other person is saying - they need to hear together what we are saying’.

The physicians also described an asymmetrical relationship in which they were the experts. One said the following:

‘Relationship and communication are more important than the patient should choose his treatment’.

The physicians assessed that patients and relatives did not have the prerequisites to make decisions about medical treatment choices. It could be stressful for the patient and relatives if the physician had expectations of user participation. The physicians emphasized that patients and relatives in such situations needed experienced physicians to guide them in the course of treatment.

Finally, the physicians were concerned with taking appropriate responsibility when choosing the course of treatment. As mentioned, they were the experts in this context, and therefore expected to assess the consequences of these choices. As patients and their relatives often experience crisis during this time, the physicians highlighted that any conveyed information must be adapted to their specific needs. Practically, they must be informed about the disease, the expected course of the disease, and the nature of required treatment during each phase of the pathway. The physicians said it was important to provide security for patients and their relatives while laying a foundation for appropriate collaboration.

Communication about the death process

The physicians said that patients and their relatives continually needed information, but that recognition was a gradual process. One said the following:

For peer review only - http://bmjopen.bmj.com/site/about/guidelines.xhtml

‘We have to take the challenge of asking the difficult questions, and we have to choose the right time to do it. I do not think everyone in the early phases has a realisation that allows us to ask how they want to die’.

In addition, the physicians conveyed that many patients had ambivalent relationships with death; at times, they would accept their situation, but only a day later express their intent to survive. However, they also said that patients should be allowed to have this dual mindset. One said the following:

‘If we're going to come in and directly inform about prognosis and ask what they want, how they want to die, then I think it's scary – it’s too brutal’.

They conveyed it was important to find the right time to provide patients with information about how their diseases would develop; that is, when the patients were ready to receive this information. As such, the physicians expressed that they spent substantial amounts of time mapping health literacy among patients and their relatives. In their experiences, there were various prerequisites for adapting to situations and processing the nature of disease development at the individual level. Patient-centred care was also a recurring theme. Here, the physicians reflected on the importance of avoiding situations in which their professional values, e.g., expectations of mastery and a ‘good death’, prevented them from handling difficult situations and enduring ethical dilemmas. In particular, they expressed the need to respect each family’s choices. One physician said the following:

‘We have to accept the situations in which we do not achieve our treatment goals or a “good death,” which is a value for us. Ain’t no cure for death’.

For peer review only - http://bmjopen.bmj.com/site/about/guidelines.xhtml

Processing relatives' guilt and grief

In the terminal phase, the physicians said the conflict between efficiency and quality constituted an ethical dilemma. For example, it could be difficult to decide how many days a patient should stay at the hospital. They said that demands for efficiency were not always in accordance with the patient's need for time during the process. In this regard, one said the following:

'Once we have made a plan, it is just to go home. But, it would have been beneficial with another day to talk through the situation, or to bring in the family'.

This was a recurrent ethical challenge experienced by physicians working in hospitals. The terminal phase also entailed the need for closer collaboration with the relatives of patients, some of whom believed it was meaningful to have more responsibility in caring for their loved ones over the death process, as this provided time and space for saying goodbye and experiencing community. Indeed, the physicians said it was important to listen to the needs of patients and their relatives while helping them achieve peace. In this context, it was often beneficial to withdraw from the process some, as patients and their relatives were the true focus. Later, the physicians said they had positive experiences when holding conversations with individuals experiencing bereavement. One said the following:

'There is not much that needs to be done, a review of the medical history and what actually happened'.

The physicians also said it was important to provide the relatives with stories they could accept over the course of their own lives. Many relatives carried guilt over whether they had done enough, e.g., that the disease was not detected early enough, or that they had not contributed enough to help care for the patient. During bereavement, conversations were thus focused on summarising what

For peer review only - http://bmjopen.bmj.com/site/about/guidelines.xhtml

occurred over the palliative pathway; the physicians said this helped the relatives end conversations with 'square shoulders'. The atmosphere was often positive, with relatives expressing gratitude and relief. The physicians felt it was important for relatives to talk about any emotional strain they had experienced during the palliative care process. The mourning process was also more conducive in cases where the relatives had made contact with physicians early in the process, and thus held continuous dialogues.

DISCUSSION

The narratives showed different needs during different phases of the palliative care process. In the early phase, the physicians conveyed that the patients and their relatives were in crises. Here, they believed it was important to provide relevant information while working to develop a common understanding. In the middle phase, the physicians experienced that the patients and their relatives continually needed information. However, timing was essential based on the type of information provided. This also made it important to map individual health literacy. Following the terminal phase, bereaved relatives needed memories that could help them in accepting what they had gone through. As such, conversations during bereavement helped the mourning process and assuaged feelings of guilt and grief.

Trusting communication

The physicians said that patients and their families experienced difficulty when transitioning from the curative to palliative phase, which made it important to communicate with all present. In palliative care, communication entails the need to develop a common understanding about what will occur. Concerning treatment situations, previous studies have emphasised that patients and their relatives depend on expert knowledge from physicians, as the doctor-patient relationship is asymmetrical due to professional roles and other factors,[5, 22] this makes trust a central component.[23] Physicians must therefore consider issues and needs from the patient's perspective, as they have entrusted their care 'in good faith'. In this study, the physicians said their patients conferred trust when allowing them to

For peer review only - http://bmjopen.bmj.com/site/about/guidelines.xhtml

decide the best course of treatment over the palliative pathway. In other words, patients assume that physicians will act in their best interests. In turn, this creates the space that physicians need to fully use their professional competencies. While this forms a basis for exercising benign power, it is also important to remember that such trust places the patient in a position of vulnerability to negative forms of power.[22] On the other hand, uncertainty about the prognosis may hinder communication between physicians when attempting to provide information about palliative treatment,[7] which likely imposes a variety of other communicative difficulties. In sum, clear communication is necessary for developing trust during vulnerable health situations. [7, 8, 22]

Compassionate communication

We found that timing was important when providing information about disease prognosis. The physicians also said they needed to ask difficult questions at the right time, which required an understanding of individual characteristics, including various levels of health literacy among patients and their relatives. Further, the physicians said they did not always achieve their intention of facilitating 'a good death' for the patient, as it was considered more important to respect and comply with the wishes of patients and their families. Indeed, compassion is widely considered an essential element in palliative care. [12, 13, 24] From the patient's perspective on palliative care, a previous study even reported that compassion and empathy were preferred over sympathy, with compassion being the most preferred, as it benefited communication that addressed their suffering and needs through relational understanding and action.[8] This definition also emphasises action as an important aspect of compassion, which reflects the practices implemented by this study's the physicians, who prioritised the preferences of patients and their relatives over their own professional values during care. Other studies have also highlighted the importance of tailored, clear, and patient-centred communication in facilitating death.[9, 14] Further, cultural competence is highly important when adapting information based on various levels of health literacy among patients and their relatives.[9] In this study, physicians also said it was important to map health literacy. Personal health literacy is

For peer review only - http://bmjopen.bmj.com/site/about/guidelines.xhtml

defined as the ability to find, understand, and use information and services to make health-related decisions and actions for themselves and others.[25]

Following the conclusion of the terminal phase, physicians said it was important to communicate during bereavement, as relatives needed stories they could accept over the course of their own lives. In the mourning process, it was helpful when physicians had been involved from early on, and were thus able to hold continual dialogues with the relatives. However, many are not given the opportunity to hold conversations with physicians when experiencing bereavement, although research has shown they believe this would have helped them deal with sorrow and loss.[26] In sum, both the current and previous findings emphasise that conversations during bereavement are important aspects of the palliative care process.

Strengths and limitations

The qualitative interview approach provided rich and complex data, as we employed an explicit theoretical framework to reveal theoretical perspectives for analysis, thus enhancing trustworthiness. Further, direct quotations underpinned the main findings.[18] The researchers also had different professional backgrounds (RN and social scientists), which aided in understand the complexity of the obtained narratives. Such intersubjective understanding also improved the study's authenticity.[17] However, the study was solely conducted in the Norwegian context. Nevertheless, research suggests that our findings may be relevant in other cultural contexts such as New Zealand, England and USA.[10, 27]

Implications of results for practice and further research

In this study, the narrative approach revealed new insights into how physicians communicate with patients and their relatives during different phases of palliative care. In practice, these findings should help improve the overall quality of communication. A close collaboration with relatives in the terminal phase is important for achieving a 'good death'. Additional studies should continue to investigate the issue with cultural dimension of communication in palliative care. It is also of interest

For peer review only - http://bmjopen.bmj.com/site/about/guidelines.xhtml

to study communication in team working between health professionals in palliative care. The digital revolution opens up new ways of communicating and it would be of great interests to explore possibilities of interacting in palliative care.

Conflict of interests

The authors report no conflicts of interests.

Funding

No funding to report.

Acknowledgment

We express our gratitude to all participating physicians who shared their experiences about communication over the palliative pathway.

Contributorship statement

Conceptualization, BJL and MK; methodology, BJL and MK; validation, BJL and MK; formal analysis, BJL and MK; investigation, BJL and MK; writing - original draft preparation, BJL and MK; writing - review and editing, BJL and MK. Both authors have read and agreed to the published version of the manuscript.

For peer review only

Ethics statement

According to the Regional Committee on Medical and Health Research Ethics, this study did not require approval (xx). However, we did receive approval from the Data Protection Officer associated with the xx (xx) and the Norwegian Centre for Research Data (xx).

Data Sharing Statement

The interviews are transcribed verbatim in specific Norwegian dialects and it is therefore not appropriate to share the data with other researchers. The dialects are very different from the two Norwegian official languages *Bokmål* and *Nynorsk* and therefore not appropriate to share.

REFERENCES

1. World Health Organization (WHO). Palliative care. https://www.who.int/news-room/fact-sheets/detail/palliative-care (accessed 1 May 2022).

2. Kaasa S, Loge JH, Aapro M, et al. Integration of oncology and palliative care: a Lancet Oncology Commission. *Lancet Oncol* 2018; 19: e588–653.

3. Cain C, Surbone A, Elk R, et al. Culture and palliative care: preferences, communication, meaning, and mutual decision making. *J Pain Symptom Manage* 2018; 55: 1408e1419.

4. Oxford Learner's Dictionaries. Communication. https://www.oxfordlearnersdictionaries.com/definition/english/communication?q=communication (accessed 1 May 2022).

5. Madssen K-A. Språk og kommunikasjon: Myndiggjering eller umyndiggjering? Kap. 9. In: Ekeland TJ and Heggen K (eds) *Meistring og myndiggjering. Reform eller retorikk?* Oslo: Gyldendal

For peer review only - http://bmjopen.bmj.com/site/about/guidelines.xhtml

Akademisk, 2007.

6. Guldbrandsen P, Fugellie P, Stang G, et al. *Skam i det medisinske rom.* Oslo: Gyldendal akademisk. 2006.

7. Udo C, Lövgren M, Lundquist G, et al. Palliative care physicians' experiences of end-of-life communication: a focus group study. *Eur J Cancer Care* 2018; 27: e12728.

8. Sinclair S, Beamer K, Hack TF, et al. Sympathy, empathy, and compassion: a grounded theory study of palliative care patients' understandings, experiences, and preferences. *Palliat Med* 2017; 31(5): 437–447.

9. Anderson R, Bloch S, Armstrong M, et al. Communication between healthcare professionals and relatives of patients approaching the end-of-life: a systematic review of qualitative evidence. *Palliat Med* 2019; 33(8): 926–941.

10. Kidd J, Black S, Blundell R, et al. Cultural health literacy: the experiences of Māori in palliative care. *Glob Health Promot* 2018; 25(4): 15–23.

11. Lavoie M, Blondeau D and Martineau I. The integration of a person-centered approach in palliative care. *Palliat Support Care* 2013; 11: 453–464.

12. Anonymous

13. Mills J, Wand T and Fraser JA. Palliative care professionals' care and compassion for self and others: a narrative review. *Int J Palliat Nurs* 2017; 23(5): 219-229.

14. Schellinger SE, Anderson EW, Frazer, MS, et al. Patient self-defined goals: essentials of person-centered care for serious illness. *Am J Hosp Palliat Med* 2018; 35(1): 159-165.

15. Clandinin DJ and Connelly FM. *Narrative inquiry: experience and story in qualitative research.* San Francisco: Jossey-Bass, 2000.

16. Patton MQ. *Qualitative research & evaluation methods: integrating theory and practice.* Los Angeles, California: Sage, 2015.

17. Holloway I and Freshwater H. *Narrative research in nursing.* Oxford, UK: Blackwell, 2007.

18. Tong, A., Sainsbury, P., & Craig, J. (2007). Consolidated criteria for reporting qualitative research

For peer review only - http://bmjopen.bmj.com/site/about/guidelines.xhtml

(COREQ): a 32-item checklist for interviews and focus groups. International journal for quality in health care, 19(6), 349-357. doi:10.1093/intqhc/mzm042

19. Morse JM and Richards L. *Read me first for a user's guide to qualitative methods*. Thousand Oaks, California: Sage, 2002.

20. Chase SE (2018) Narrative Inquiry Toward Theoretical and Methodological Maturity. In: Denzin NK and Lincoln YS (eds) The Sage handbook of qualitative research. 5 ed. Los Angeles, Calif: Sage

21. Brinkmann S and Kvale S. *InterViews: learning the craft of qualitative research interviewing.* Thousand Oaks, Calif: Sage. 2015.

22. Grimen H. *Hva er tillit? (what is trust?)* Oslo: Univeristetsforlaget, 2009.

23. Grimen H. Profesjon og tillit (Profession and trust). In: Molander A and Terum LI (eds) *Profesjonsstudier (vocational studies)* Oslo: Universitetsforlaget, 2008, pp.197–215.

24. Anonymous

25. What Is Health Literacy? https://www.cdc.gov/healthliteracy/learn/index.html (accessed 1 May 2022).

26. Anonymous

27. Seymour J, Cassel. Palliative care in the USA and England: a critical analysis of meaning and implementation towards a public health approach, *Mortality 2017*, 22:4, 275-290, DOI: 10.1080/13576275.2016.1270262.

For peer review only - http://bmjopen.bmj.com/site/about/guidelines.xhtml

Additional file 1 Standards for Reporting Qualitative Research (SRQR)

Topic	Page/line no(s).
**Title and abstract**	
**Title** - Concise description of the nature and topic of the study Identifying the study as qualitative or indicating the approach (e.g., ethnography, grounded theory) or data collection methods (e.g., interview, focus group) is recommended	1
**Abstract** - Summary of key elements of the study using the abstract format of the intended publication; typically includes background, purpose, methods, results, and conclusions	2
**Introduction**	
**Problem formulation** - Description and significance of the problem/phenomenon studied; review of relevant theory and empirical work; problem statement	4-5
**Purpose or research question** - Purpose of the study and specific objectives or questions	5
**Methods**	
**Qualitative approach and research paradigm** - Qualitative approach (e.g., ethnography, grounded theory, case study, phenomenology, narrative research) and guiding theory if appropriate; identifying the research paradigm (e.g., postpositivist, constructivist/ interpretivist) is also recommended; rationale**	5
**Researcher characteristics and reflexivity** - Researchers' characteristics that may influence the research, including personal attributes, qualifications/experience, relationship with participants, assumptions, and/or presuppositions; potential or actual interaction between researchers' characteristics and the research questions, approach, methods, results, and/or transferability	14-15
**Context** - Setting/site and salient contextual factors; rationale**	5-6
**Sampling strategy** - How and why research participants, documents, or events were selected; criteria for deciding when no further sampling was necessary (e.g., sampling saturation); rationale**	6
**Ethical issues pertaining to human subjects** - Documentation of approval by an appropriate ethics review board and participant consent, or explanation for lack thereof; other confidentiality and data security issues	8
**Data collection methods** - Types of data collected; details of data collection procedures including (as appropriate) start and stop dates of data collection and analysis, iterative process, triangulation of sources/methods, and modification of procedures in response to evolving study findings; rationale**	7
**Data collection instruments and technologies** - Description of instruments (e.g., interview guides, questionnaires) and devices (e.g., audio recorders) used for data collection; if/how the instrument(s) changed over the course of the study	7
**Units of study** - Number and relevant characteristics of participants, documents, or events included in the study; level of participation (could be reported in results)	6
**Data processing** - Methods for processing data prior to and during analysis, including transcription, data entry, data management and security, verification of data integrity, data coding, and anonymization/de-identification of excerpts	7
**Data analysis** - Process by which inferences, themes, etc., were identified and developed, including the researchers involved in data analysis; usually references a specific paradigm or approach; rationale**	8
**Techniques to enhance trustworthiness** - Techniques to enhance trustworthiness and credibility of data analysis (e.g., member checking, audit trail, triangulation); rationale**	14-15
**Results/findings**	
**Synthesis and interpretation** - Main findings (e.g., interpretations, inferences, and themes); might include development of a theory or model, or integration with prior research or theory	8-12
**Links to empirical data** - Evidence (e.g., quotes, field notes, text excerpts, photographs) to substantiate analytic findings	12-15
**Discussion**	
**Integration with prior work, implications, transferability, and contribution(s) to the field** - Short summary of main findings; explanation of how findings and conclusions connect to, support, elaborate on, or challenge conclusions of earlier scholarship; discussion of scope of application/generalizability; identification of unique contribution(s) to scholarship in a discipline or field	14
**Limitations** - Trustworthiness and limitations of findings	14-15
**Other**	
**Conflicts of interest** - Potential sources of influence or perceived influence on study conduct and conclusions; how these were managed	N/A
**Funding** - Sources of funding and other support; role of funders in data collection, interpretation, and reporting	N/A

For peer review only - http://bmjopen.bmj.com/site/about/guidelines.xhtml

*The authors created the SRQR by searching the literature to identify guidelines, reporting standards, and critical appraisal criteria for qualitative research; reviewing the reference lists of retrieved sources; and contacting experts to gain feedback. The SRQR aims to improve the transparency of all aspects of qualitative research by providing clear standards for reporting qualitative research.

**The rationale should briefly discuss the justification for choosing that theory, approach, method, or technique rather than other options available, the assumptions and limitations implicit in those choices, and how those choices influence study conclusions and transferability. As appropriate, the rationale for several items might be discussed together.

Reference:
O'Brien BC, Harris IB, Beckman TJ, Reed DA, Cook DA. **Standards for reporting qualitative research: a synthesis of recommendations.** *Academic Medicine*, Vol. 89, No. 9 / Sept 2014
DOI: 10.1097/ACM.0000000000000388

For peer review only

For peer review only - http://bmjopen.bmj.com/site/about/guidelines.xhtml

BMJ Open

Physicians' narratives of communication with patients and their relatives in different phases of the palliative pathway

Journal:	*BMJ Open*
Manuscript ID	bmjopen-2022-065681.R1
Article Type:	Original research
Date Submitted by the Author:	11-Apr-2023

Complete List of Authors:	Landstad, Bodil J.; Mid Sweden University Faculty of Human Science, Health Sciences; Östersund Hospital, Unit of Research, Education and Development<br>Kvangarsnes, Marit; Norwegian University of Science and Technology; Møre og Romsdal Hospital Trust

**Primary Subject Heading**:	Palliative care
Secondary Subject Heading:	Communication, General practice / Family practice, Health services research, Palliative care, Qualitative research
Keywords:	Quality in health care < HEALTH SERVICES ADMINISTRATION & MANAGEMENT, Adult palliative care < PALLIATIVE CARE, QUALITATIVE RESEARCH

SCHOLARONE™
Manuscripts

I, the Submitting Author has the right to grant and does grant on behalf of all authors of the Work (as defined in the below author licence), an exclusive licence and/or a non-exclusive licence for contributions from authors who are: i) UK Crown employees; ii) where BMJ has agreed a CC-BY licence shall apply, and/or iii) in accordance with the terms applicable for US Federal Government officers or employees acting as part of their official duties; on a worldwide, perpetual, irrevocable, royalty-free basis to BMJ Publishing Group Ltd ("BMJ") its licensees and where the relevant Journal is co-owned by BMJ to the co-owners of the Journal, to publish the Work in this journal and any other BMJ products and to exploit all rights, as set out in our licence.

The Submitting Author accepts and understands that any supply made under these terms is made by BMJ to the Submitting Author unless you are acting as an employee on behalf of your employer or a postgraduate student of an affiliated institution which is paying any applicable article publishing charge ("APC") for Open Access articles. Where the Submitting Author wishes to make the Work available on an Open Access basis (and intends to pay the relevant APC), the terms of reuse of such Open Access shall be governed by a Creative Commons licence – details of these licences and which Creative Commons licence will apply to this Work are set out in our licence referred to above.

Other than as permitted in any relevant BMJ Author's Self Archiving Policies, I confirm this Work has not been accepted for publication elsewhere, is not being considered for publication elsewhere and does not duplicate material already published. I confirm all authors consent to publication of this Work and authorise the granting of this licence.

For peer review only

Title of the article

Physicians' narratives of communication with patients and their relatives in different phases of the palliative pathway

Authors

1. LANDSTAD Bodil J.*
    a. Faculty of Human Sciences, Mid Sweden University, Sweden
    b. Unit of Research, Education and Development, Östersund Hospital, Östersund, Sweden

    Address: Faculty of Human Sciences, Mid Sweden University, Campus Östersund, 831 25 Östersund, Sweden

    E-mail: bodil.landstad@miun.se

    Phone: +46 730912884

2. KVANGARSNES Marit
    a. Department of Health Sciences, Norwegian University of Science and Technology, Ålesund, Norway
    b. Møre og Romsdal Hospital Trust, Ålesund, Norway

*Corresponding author

Keywords

Physicians, qualitative research, narrative inquiry, communication, palliative care, clinical pathway

Word count

4 342 (excluding title page, abstract, references)

For peer review only - http://bmjopen.bmj.com/site/about/guidelines.xhtml

Abstract

Objectives: To explore physicians' experiences of the communication with patients and their relatives in the different phases of the palliative pathway.

Methods: Purposeful sampling was employed to recruit a total of 13 oncologists and general practitioners who engaged in palliative care. A qualitative study with a narrative approach was conducted. Interviews with physicians working in primary and specialist health care were conducted via Skype Business in the spring of 2020. The interview guide had open-ended questions with each interview lasting between 35 and 60 minutes.

Results: Communication between the physicians, their patients and their relatives was contextual and changed depending on the phase in the palliative pathway. In the first phase, physicians told us that patients and their relatives experienced an emotional shock. Transitioning from the curative to palliative phase was difficult, which emphasised the need for trust through communication. The middle phase, they revealed that communication about the death process became the priority: what was probably going to happen, the family's role in what was going to happen and perhaps, depending on the illness, any medical decisions that needed to be made. It was important for the physicians to communicate information about the palliative pathway while providing the relatives with knowledge that facilitated any decision-making. In the terminal phase physicians employed a compassionate approach, as bereaved family members needed to process their feelings of guilt and grief.

Conclusions: The study gives new insight into communication with patients and their relatives during different phases of the palliative pathway, from the physician's perspective. The findings may help physicians improve the quality of communication with patients and their relatives over these vulnerable pathways. The findings also have practical implications in training contexts. The study reveals ethical dilemmas in physician's communication with patients and their relatives during a palliative pathway.

For peer review only

Strengths and limitations of this study

- The narrative approach was appropriate when analysing communication in the different phases of the palliative pathway
- The theoretical framework gave new insights into patterns of communication that occurs in the different phases
- The researchers had different professional backgrounds
- The results are transferable to settings with similar health services

INTRODUCTION

Health care provider's communication with patients and their relatives in palliative care is crucial.[1] Patients and relatives are vulnerable during this time. A study from New Zealand found the emotional shock and grief that comes with learning of a terminal illness makes it very difficult to hear and process the given health information.[2] Empathy, both as a desirable virtue and as a communication process, has been highlighted in earlier research. Knowing and understanding the patient's perspective can assist clinicians in supporting and guiding the patient through their cancer trajectory.[3]

Communication deficits cause unnecessary distress for both patients and their relatives.[4, 5] The majority of formal complaints in health care are related to communication.[4, 6, 7] Palliative care with patients who have limited health literacy poses particular demands on communication.[8] A Swedish study researching experiences in end-of-life communication showed that there existed a communication barrier when the physicians were uncertain about prognoses, which possibly hindered treatment decisions.[9] The complex nature of the transition from curative to palliative treatment can instill uncertainty and therefore diminish the potential for good dialogues.[9]

For peer review only - http://bmjopen.bmj.com/site/about/guidelines.xhtml

In this study, we used the definition for palliative care provided by the World Health Organization (WHO). Palliative care is an approach that improves the quality of life of patients and their families facing problems associated with a life-threatening illness. Although palliative care is seen as an ethical responsibility, global estimates show that only 14% of patients in need actually receive it.[1] It has been shown to reduce suffering through the early identification, correct assessment and treatment of pain and other problems, whether physical, psychosocial or spiritual'.[1] Palliative care is thus an interdisciplinary approach in which physicians play an important role. As such, this study focused on communication with patients and their relatives by soliciting narratives of communication from the physician's perspective, as this is a highly important component for ensuring quality care.[10] In this context, communication refers to the interpersonal activity of expressing ideas and feelings or providing information; as derived from Latin, communication also refers to 'something you have in common'.[11]

Time and space create the contextual conditions for communication,[12] in which case the physical framework also serves as an important basis. This investigation focused on the type of communication that occurs between physicians, their patients and the patient's relatives in hospitals, home environments, or municipal health service institutions. It is an asymmetrical form of communication with a clear purpose. Here, the patient is in a vulnerable position due to both their dependence on the physician's competence and their access to treatment.[12] As a result of this vulnerability, any such communication is linked to the physician's professional role and position within the specific system, making symmetrical communication undesirable and unprofessional. The relative power held by the physician in this role also entails the risk that patients and their relatives will experience dependency and shame.[13] Research has shown that many patients experience shame, guilt and shortcoming in the communication with the physicians in the treatment process.[13]

For peer review only - http://bmjopen.bmj.com/site/about/guidelines.xhtml

For peer review only

Research has shown that in acute and critical situations, the need for shared decision-making is reduced.[14, 15] Trust, therefore, is a central concept in communication between physicians, the patient and their relatives during the palliative pathway. Physicians have both theoretical and practical knowledge whereas patients and relatives usually have neither; therefore, patients are often required to bestow their trust to physicians for decisions on treatment. The patients presuppose that the physician will act in their best interest. This kind of trust-based relationship is, from the physician's side, ultimately necessary to create the needed space to fully utilize their professional competence.[16]

Sympathy, empathy, and compassion are all important for patients at the end stage of life, but research has shown that patients place the highest value on compassion.[17] A systematic review of 31 studies aimed at communication between healthcare professionals and the relatives of patients approaching the end-of-life stage identified seven relevant themes including highlighting deterioration, involvement in decision-making, post-decision interactional work, tailoring, honesty and clarity, specific techniques for information delivery, and the roles of different healthcare professionals in communication.[18] Patient-centered care and compassion is paramount for patients and relatives during communication in palliative care.[19, 20, 21, 22]

Understanding the patient's level of medical knowledge and understanding, i.e., their health literacy, as well as any other factors that could impact the patient's ability to understand their situation is critical for the physician to accurately relay information. An example of the consequences of not tailoring information to ensure that patients understand was shown in a study of cultural knowledge and understanding in the medical field, which found that a lack of cultural understanding among health professionals led to late access or the avoidance of palliative care among indigenous peoples.[2]

For peer review only

The objective of the study is to explore physicians' communication with patients and their relatives in the different phases of the palliative pathways: the early, middle and, terminal/bereavement phases. We defined these phases as the initial days following the diagnosis of an incurable disease (the early phase), the time between the early phase and the last weeks, or days leading up to death (the middle phase), and the last weeks before death and the time after death (terminal/bereavement phase).[23] We therefore posed specific questions in the interview guide to assess the use of communication in the different phases. There is a lack of knowledge about communication in different phases of palliative care from the physician's perspective.

METHODS

The aim of the study was to explore physicians' experiences of the communication with patients and their relatives in the different phases of the palliative pathway. The study has the following research question: How do physicians experience communication with patients and relatives in different phases of the palliative pathway? We employed a narrative approach and subsequent analysis, which entails different theoretical directions that capture personal and human dimensions of experience over time by considering the relationship between individual experiences and cultural contexts.[24] Here, the narrative approach concerned how physicians perceived communication in the early, middle, and terminal/bereavement phases of palliative pathway based on interview data.[25, 26] Narrative analysis emphasizes the physicians voice in different phases of the palliative pathway.[26] The study was compiled with the consolidated criteria for reporting qualitative research (COREQ).[27]

Study population and recruitment

The physicians were recruited by purposive sampling aimed at capturing the complexity of the palliative pathway.[25, 28] The inclusion criteria were physicians with experience from all three phases of care defined in this study.

For peer review only - http://bmjopen.bmj.com/site/about/guidelines.xhtml

A contact person recruited a total of 13 physicians working in palliative care, using a direct approach for recruitment through cold calling or face-to-face contact. To ensure diversity and robustness, we recruited males and females of various age groups, experience levels, and medical backgrounds.

Data collection

We developed an interview guide (Table 1) with open-ended questions based on the study's aim and previous research.[29] This facilitated the solicitation of personal experiences related to communication through the progression of palliative pathway.[30] More specifically, the questions were related to how physicians perceived communication during the different phases of the palliative pathway.

Table 1. Interview guide.

	**Questions**
1	How did you experienced being a physician in palliative care?
2	What is important in communication with patients and relatives in the early phase of the illness?
3	Can you tell about your communication in the middle/terminal phase of the pathway?
4	How do you interact with patients and relatives?
5	What is of importance in communication in the bereavement phase?
6	Is there anything else you want to share with us?

Individual narrative interviews were conducted via Skype Business between April and May 2020. Each interview lasted between 35 and 60 minutes. The interviewer was a researcher as well as cancer nurse, trained to conduct interviews.[27] Saturation was reached upon observing that the data gathered through subsequent interviews yielded redundant information.[25] After the 13 interviews, the data were relating to the different phases of the palliative pathway.

For peer review only - http://bmjopen.bmj.com/site/about/guidelines.xhtml

Patient and public involvement

None

Data analysis

All interviews were audio-recorded and transcribed verbatim. These transcripts were then read to gain an overview of the data.[29] The result section contains stories from 13 physicians that form one narrative. A narrative inquiry research method was chosen as it considers lived experiences and allows for the events that have been of most significance to be brought forth in the stories participants tell. The interviewed physicians had rich and thick experiences from all phases of the palliative pathway. A narrative does not represent "the truth". In narrative research the researchers are co-creators in shaping the narrative. A narrative is formed by the way we give meaning to our experiences. This means how we understand our own or others' actions as well as the way we organise the events in our minds. How we connect and perceive the consequences of actions over time is of importance for the shaping of the narrative. Meaning making of our experiences can be linked to the past, the present, and the future.[29]

During a subsequent content analysis, we searched for a plot based on patterns in the narratives. Specifically, this plot described communication during the early, middle, and terminal phases of the palliative pathway.[25, 26]

Ethical consideration

All participants were informed about the study's purpose and procedures, in both oral and written formats. They were told that participation was voluntary and that they could withdraw at any time.

For peer review only - http://bmjopen.bmj.com/site/about/guidelines.xhtml

Further, they were informed that any obtained data would be handled confidentially. The physicians provided informed written consent.

RESULTS

Oncologists and general practitioners (GPs) who worked in hospitals or primary care settings provided information about how they supported patients and their relatives during different phases of the palliative pathway (see Table 2).

Table 2. Demographic data on the study participants.

	**Participants** (N=13)
**Gender**	
Men	10
Women	3
**Age**	
41-50	7
51-60	4
61-70	2
**Place of assignment***	
Hospital	7
Primary care	7
Private clinic	1
**Education****	
General practitioner	5
Oncologist	5
Physician	5
**Years of experience**	

For peer review only

10-15	2
16-20	5
21-25	-
26-30	4
>30	2

*Some physicians reported more than one workplace.

**Some physicians worked as both oncologists and general practitioners.

For peer review only

We derived the following three themes related to communication in the 1) early, 2) middle, and 3) terminal/bereavement phases, respectively: 1) trusting communication, 2) communication about the death process, and 3) communication about processing relatives' guilt and grief. In Table 3 we present the analysis process for all three themes. The themes illustrate what the physicians considered as significant in the professional communication with patients and relatives in the different phases of the palliative pathway.

Table 3. The analysis process for the three themes.

Quotation	Subtheme	Main theme
Relationship and communication are more important than the patient's choice of treatment	Paternalistic communication	
We should take the challenge of talking to patients and relatives together, because they need to hear what the	Joint communication	**Trusting communication**

other person is saying - they need to hear together what we are saying		
Any conveyed information must be adapted to their specific needs	Adapted communication	
We should take the challenge of talking to patients and relatives together, because they need to hear what the other person is saying - they need to hear together what we are saying.	Common understanding	**Communication about the death process**
If we're going to come in and directly inform about prognosis and ask what they want, how they want to die, then I think it's scary - it's too brutal.	Compassion	
We have to accept [situations in which] we do not achieve our treatment goals or a 'good death', which is a value for us. Ain't no cure for death.	Respect for the choices of patients and their relatives	

… it would have been beneficial with another day to talk through the situation, or to bring in the family.	To talk through the situation with the family	**Communication about processing relatives' guilt and grief**
There is not much that needs to be done, a review of the medical history and what actually happened.	Listening to the families experiencing bereavement	
It was important to provide the relatives with stories they could accept and carry with them when going forward.	To give relatives stories to live with	

1. Trusting communication

The physicians described an asymmetrical relationship in which they were the experts. One said the following:

'Relationship and communication are more important than the patient's choice of treatment'.

The narratives revealed that the transition from curative to palliative care as difficult. In addition to the disease-related ailments, patients and their relatives underwent an internalised process of acceptance which often entailed feelings of shock, especially for patients who now realized their limited lifespan. The transition was also often the beginning of a mental process leading to cognition and acceptance of death. Patients had to avail the resources, networks, and coping strategies they built up over the course of their lifetime. Time was marked by loss, and patients learned not expect improvements.

For peer review only - http://bmjopen.bmj.com/site/about/guidelines.xhtml

Physicians expressed they wanted to talk with patients and their relatives together. Here, the goal was to help patients and their relatives develop a common understanding of their situation and the palliative care pathway. One participant said the following:

‘We should take the challenge of talking to patients and relatives together, because they need to hear what the other person is saying - they need to hear together what we are saying’.

The physicians experienced that patients and relatives did not have enough knowledge to make decisions about medical treatment choices. The physicians emphasized that patients and relatives in such situations needed experienced physicians to guide them in the course of treatment. This was a way of protecting the patients and their relatives from difficult choices.

This presumed responsibility causes stress for the physicians, who were concerned with being responsibility for the recommended choice of treatment. Being experts in this context, physicians are therefore expected to assess the consequences of the available choices to the patient’s specific situation and relay that to the patient. As patients and their relatives often experience crisis during this time, the physicians highlighted that “any conveyed information must be adapted to their [the patient’s] specific needs”. Practically, the patients must be informed about the disease, the expected course of the disease, and the nature of the treatment during each phase of the pathway. The physicians said it was important to provide this information, by which giving security to patients and their relatives while laying a foundation for appropriate collaboration.

2. Communication about the death process

The physicians said that patients and their relatives continually needed information, but that recognition was a gradual process. One said the following:

For peer review only - http://bmjopen.bmj.com/site/about/guidelines.xhtml

‘We have to take the challenge of asking the difficult questions, and we have to choose the right time to do it. I do not think everyone in the early phases has a realisation that allows us to ask how they want to die’.

‘If we're going to come in and directly inform about prognosis and ask what they want, how they want to die, then I think it's scary – it’s too brutal’.

The physicians conveyed that many patients had ambivalent relationships with death; at times, they would accept their situation, but only a day later express their intent to survive. However, they also said that it was normal and totally acceptable for the patients to have this dual mindset.

Timing is always of essence, especially when communicating with patients, whether to inform or ask questions. Finding the right time to provide patients with information on their disease’s development is very important and that means being cognizant of their patient’s mental status and realizing when the patient is ready to receive this information. As such, the physicians expressed that they spent substantial amounts of time to get an overview of health literacy among patients and their relatives. In their experiences, there were various prerequisites for adapting to situations and processing the nature of disease development at the individual level.

Patient-centered care was also a recurring theme. Here, the physicians reflected on the importance of avoiding situations in which their professional values, e.g., expectations of mastery and a ‘good death’, prevented them from handling difficult situations and enduring ethical dilemmas. In particular, they expressed the need to respect each family’s choices. One physician said the following:

For peer review only - http://bmjopen.bmj.com/site/about/guidelines.xhtml

‘We have to accept the situations in which we do not achieve our treatment goals or a “good death,” which is a value for us. Ain’t no cure for death’.

3. Communication about processing relatives’ guilt and grief

In the terminal/bereavement phase, the physicians said the conflict between efficiency and quality constituted an ethical dilemma in which the demands for efficiency were not always in accordance with the patient’s need for time during the process. One example, noted by a physician, was determining how long a patient should stay at the hospital, saying the following:

‘… it would have been beneficial with another day to talk through the situation, or to bring in the family’.

This recurrent ethical challenge was experienced often by physicians working in hospitals. The terminal phase entails the need for greater involvement of the relatives of patients as it often gives meaning to them during the death process. Having some of the responsibility in caring for their loved ones provided time and space for saying goodbye and experiencing community. There is an importance in listening to the needs of patients and their relatives all the while helping them through the process of acceptance. In this context, it was often beneficial for the physicians to withdraw from the process some, as the patients and their relatives were the true focus. When the process gets to certain point, the focus becomes helping the patients and family come to terms with the situation, as medically there is nothing more that can be done, noted in the following quote:

‘There is not much that needs to be done, a review of the medical history and what actually happened’.

For peer review only - http://bmjopen.bmj.com/site/about/guidelines.xhtml

The process of acceptance for the family is challenging and many relatives often carry guilt moving forward, so 'it was important to provide the relatives with stories they could accept and carry with them when going forward', to help them come to terms with the death. This guilt stems from many areas including that the disease was not detected early enough, or that they had not contributed enough. During bereavement, conversations were thus focused on summarising what occurred over the palliative pathway; the physicians said this helped the relatives end conversations with 'relaxed shoulders'. The atmosphere was often positive, with relatives expressing gratitude and relief. The physicians felt it was important for relatives to talk about any emotional strain they had experienced during the palliative care process. The mourning process was also more conducive in cases where the relatives had contact with physicians early in the process, and thus held continuous dialogues.

DISCUSSION

The narratives showed various experiences with professional communication during different phases of the palliative pathway. In the early phase, the physicians conveyed that, as the patients and their relatives were in crises mode, trustful communication was crucial. Here, they saw it as important to provide relevant information while working to develop a common understanding. In the middle phase, the need for information was strong with the patients and their families having many questions. Compassion, respect and a common understanding are considered significant in this dialog and more importantly for the physicians was the timing of the information given. Following this phase into the final and terminal phase, the bereaved relatives needed experiences, interactions and memories that could help them through the acceptance process. As such, conversations during bereavement helped the mourning process and assuaged feelings of guilt and grief.

The transitioning from the curative to palliative phase is difficult for all involved making the dialog between the physicians and the patient and their families even more important. In palliative care,

For peer review only - http://bmjopen.bmj.com/site/about/guidelines.xhtml

For peer review only

the goal of the communication is to develop a common understanding of the coming situation that will occur. This dialog is a way to create trust, which is a central component.[31]

Previous studies have emphasised that patients and their relatives depend on expert knowledge from physicians making the doctor-patient relationship asymmetrical due to professional roles and other factors.[12, 16] Research has shown that in acute and critical situations, the need for shared decision-making is reduced.[14, 15] Physicians must therefore consider issues and needs from the patient's perspective, as they are entrusted with the care of the patient 'in good faith'. In this study, the physicians said their patients conferred trust when allowing them to decide the best course of treatment over the palliative pathway. In other words, patients assume that physicians will act in their best interests. In turn, this creates the space that physicians need to fully use their professional competencies. While this forms a basis for exercising benign power, it is also important to remember that such trust places the patient in a position of vulnerability to negative forms of power.[16] On the other hand, uncertainty about the prognosis may hinder communication between physicians when attempting to provide information about palliative treatment,[9] which likely imposes a variety of other communicative difficulties. In sum, clear communication is necessary for developing trust during vulnerable health situations.[9, 17, 16]

We found that timing was important when providing information about disease prognosis. The physicians said they needed to ask difficult questions at the right time, which required an understanding of individual characteristics, including various levels of health literacy among patients and their relatives. The physicians said they did not always achieve their intention of facilitating 'a good death' for the patient, as it was considered important to respect and comply with the wishes of patients and their families. Compassion is widely considered an essential element in palliative care.[20, 21, 32] A previous study reported that compassion and empathy were preferred over sympathy from the perspective of the patient on palliative care.[17] Compassion, in this case, being the most preferred, as it benefited communication addressing their suffering and needs through relational understanding

For peer review only - http://bmjopen.bmj.com/site/about/guidelines.xhtml

and action.[17] This definition also emphasises action as an important aspect of compassion, which reflects the practices implemented by this study's physicians, who prioritised the preferences of patients and their relatives over their own professional values during care. Other studies have also highlighted the importance of tailored, clear, and patient-centered communication in facilitating death.[18, 22] Further, cultural competence is highly important when adapting information to the various levels of health literacy among patients and their relatives.[18] Personal health literacy is defined as the ability to find, understand, and use information and services to make health-related decisions and actions for themselves and others.[33] In this study, physicians said it was important to map the health literacy to understand the level of comprehension of the patient and their family members. In doing so, the physician was better adept to change how they spoke to those involved ensuring their message was received and understood as intended.

Following the death of the patient, physicians stated that continued communication during bereavement also important. Relatives needed stories they could relate to within their own lives. In the mourning process, it was helpful when physicians had been involved from early on and were thus able to hold continual dialogues with the relatives. However, research has shown that physicians come into the picture at a later stage in the process and many relatives are not given the opportunity to have these conversations.[32] In sum, both the current and previous findings emphasise that the continuous dialog during bereavement are important aspects of the palliative care process.

For peer review only - http://bmjopen.bmj.com/site/about/guidelines.xhtml

Conclusion

The study gives new insight into physician's experiences of communication in different phases of the palliative pathway. Trustful communication with patients and their relatives were crucial in the first phase. In the middle phase communication about the death process is important and, respect for the choices of patients and their relatives were important. A common understanding with the patient and their relatives of the treatment was paramount. In the bereavement phase, talking through the situation with the relatives and giving them stories to live with was of great significance for the physicians.

A conclusion is that the physicians may experience an ethical dilemma in communication with patients and their relatives about the efficiency and quality in the treatment. Conflicts might arise between the physicians and the relatives in, for example, treatment choices. Taking a paternalistic attitude to protect the patient from harm can be morally complicated. The ethical dilemmas physicians may experience should be an important topic of discussion in the community of practices of physicians as well as in medical education.

Strengths and limitations

This study is limited to the experiences of the physicians. No patient and public involvement is included in the study. The qualitative interview approach provided rich and complex data, as we employed a theoretical framework of communication and trust to reveal theoretical perspectives for analysis, thus enhancing trustworthiness.[12, 16, 30] Further, direct quotations underpinned the main findings.[27] The researcher's different professional backgrounds, being RN and social scientists, aided in understand the complexity of the obtained narratives. Such intersubjective understanding also improved the study's authenticity.[26] Member checking was done during the interview process by an experienced oncologist. The study was solely conducted in the Norwegian context. Nevertheless,

For peer review only - http://bmjopen.bmj.com/site/about/guidelines.xhtml

For peer review only

research suggests that our findings may be relevant in other cultural contexts such as New Zealand, Europe and USA.[2, 34]

Implications of results for practice and further research

In this study, the narrative approach revealed new insights into how physicians communicate with patients and their relatives during different phases of palliative care. In practice, these findings should help improve the overall quality of communication. A close collaboration with relatives in the terminal phase is important for achieving a 'good death'. Additional studies should continue to investigate the issue with cultural dimension of communication in palliative care. It is also of interest to study communication within the team working between health professionals in palliative care. The digital age creates new ways of communicating and it would be interesting to explore the possibilities of this interaction in palliative care.

Conflict of interests

The authors report no conflicts of interests.

Funding

No funding to report.

Acknowledgment

We express our gratitude to all participating physicians who shared their experiences about communication over the palliative pathway.

For peer review only - http://bmjopen.bmj.com/site/about/guidelines.xhtml

Contributorship statement

Conceptualization, BJL and MK; methodology, BJL and MK; validation, BJL and MK; formal analysis, BJL and MK; investigation, BJL and MK; writing - original draft preparation, BJL and MK; writing - review and editing, BJL and MK. Both authors have read and agreed to the published version of the manuscript.

Ethics statement

According to the Regional Committee on Medical and Health Research Ethics, this study did not require approval (REK 78067). However, we did receive approval from the Data Protection Officer associated with the Møre og Romsdal Hospital Trust (HMR 2020/397-2) and the Norwegian Centre for Research Data (NSD 131948).

Data Sharing Statement

The interviews are transcribed verbatim in specific Norwegian dialects and it is therefore not appropriate to share the data with other researchers. The dialects are very different from the two Norwegian official languages *Bokmål* and *Nynorsk* and therefore not appropriate to share.

REFERENCES

For peer review only - http://bmjopen.bmj.com/site/about/guidelines.xhtml

1. World Health Organization (WHO). Palliative care. https://www.who.int/news-room/fact-sheets/detail/palliative-care (accessed 1 May 2022).

2. Kidd J, Black S, Blundell R, et al. Cultural health literacy: the experiences of Māori in palliative care. *Glob Health Promot* 2018; 25(4): 15–23.

3. van Vliet LM & Back AL. The different faces of empathy in cancer care: From a desired virtue to an evidence-based communication process. Cancer 2021;127:4137-4139 https://doi.org/10.1002/cncr.33833

4. van Vliet LM, Epstein AS. The current state of the art and science of patient-clinician communication in progressive disease: Patients' need to know and need to feel known. J Clin Oncol 2014;32:3474-8 https://pubmed.ncbi.nlm.nih.gov/25267758/

5. Morita T, Akechi T, Ikenaga T, et al: Communication about the ending of anticancer treatment and transition to palliative care, Ann Oncol 15: 1551- 1557, 2004.

6. Pincock S: Poor communication lies at heart of NHS complaints, says ombudsman, BMJ, 2004, 328: 10.

7. Tamblyn R, Abrahamowicz M, Dauphinee D, et al: Physician scores on a national clinical skills examination as predictors of complaints to medical regulatory authorities, JAMA, 2007, 298: 993- 1001.

For peer review only - http://bmjopen.bmj.com/site/about/guidelines.xhtml

8. Roodbeen R, Vreke A, Boland G, Rademakers J, van den Muijsenbergh M, Noordman J, et al. (2020) Communication and shared decision-making with patients with limited health literacy; helpful strategies, barriers and suggestions for improvement reported by hospital-based palliative care providers. PLoS ONE 15(6): e0234926. https://doi.org/10.1371/journal.pone.0234926

9. Udo C, Lövgren M, Lundquist G, et al. Palliative care physicians' experiences of end-of-life communication: a focus group study. *Eur J Cancer Care* 2018; 27: e12728.

10. Cain C, Surbone A, Elk R, et al. Culture and palliative care: preferences, communication, meaning, and mutual decision making. *J Pain Symptom Manage* 2018; 55: 1408e1419.

11. Oxford Learner's Dictionaries. Communication. https://www.oxfordlearnersdictionaries.com/definition/english/communication?q=communication (accessed 1 May 2022).

12. Madssen K-A. Språk og kommunikasjon: Myndiggjering eller umyndiggjering? Kap. 9. In: Ekeland TJ and Heggen K (eds) *Meistring og myndiggjering. Reform eller retorikk?* Oslo: Gyldendal Akademisk, 2007.

13. Guldbrandsen P, Fugellie P, Stang G, et al. *Skam i det medisinske rom.* Oslo: Gyldendal akademisk. 2006.

14. Bårdsgjerde EK, Kvangarsnes M, Landstad B, Nylenna M, Hole T. Patients' narratives of their patient participation in the myocardial infarction pathway. J Adv Nurs. 2019;75:1063-1073. https://doi.org/10.1111/jan.13931

For peer review only - http://bmjopen.bmj.com/site/about/guidelines.xhtml

15. Thompson, A. G. H. (2007). The meaning of patient involvement and participation in health care consultations: A taxonomy. Social Science & Medicine, 64(6), 1297–1310. https://doi.org/10.1016/j.socscimed. 2006.11.002

16. Grimen H. *Hva er tillit? (What is trust?)* Oslo: Univeristetsforlaget, 2009.

17. Sinclair S, Beamer K, Hack TF, et al. Sympathy, empathy, and compassion: a grounded theory study of palliative care patients' understandings, experiences, and preferences. *Palliat Med* 2017; 31(5): 437–447.

18. Anderson R, Bloch S, Armstrong M, et al. Communication between healthcare professionals and relatives of patients approaching the end-of-life: a systematic review of qualitative evidence. *Palliat Med* 2019; 33(8): 926–941.

19. Lavoie M, Blondeau D and Martineau I. The integration of a person-centered approach in palliative care. *Palliat Support Care* 2013; 11: 453–464.

20. Crawford P, Brown B, Kvangarsnes M, et al. The design of compassionate care. *J Clin Nurs* 2014; 23(23-24): 3589-99.

21. Mills J, Wand T and Fraser JA. Palliative care professionals' care and compassion for self and others: a narrative review. *Int J Palliat Nurs* 2017; 23(5): 219-229.

22. Schellinger SE, Anderson EW, Frazer, MS, et al. Patient self-defined goals: essentials of person-centered care for serious illness. *Am J Hosp Palliat Med* 2018; 35(1): 159-165.

For peer review only - http://bmjopen.bmj.com/site/about/guidelines.xhtml

23. Tarberg Skorpen A, Kvangarsnes M, Hole T, et al. Silent voices – family caregivers' narratives of palliative pathways. *Nurs Open* 2019; 00: 1–9.

24. Clandinin DJ and Connelly FM. *Narrative inquiry: experience and story in qualitative research.* San Francisco: Jossey-Bass, 2000.

25. Patton MQ. *Qualitative research & evaluation methods: integrating theory and practice.* Los Angeles, California: Sage, 2015.

26. Holloway I and Freshwater H. *Narrative research in nursing.* Oxford, UK: Blackwell, 2007.

27. Tong, A., Sainsbury, P., & Craig, J. (2007). Consolidated criteria for reporting qualitative research (COREQ): a 32-item checklist for interviews and focus groups. International journal for quality in health care, 19(6), 349-357. doi:10.1093/intqhc/mzm042

28. Morse JM and Richards L. *Read me first for a user's guide to qualitative methods*. Thousand Oaks, California: Sage, 2002.

29. Chase SE (2018) Narrative Inquiry Toward Theoretical and Methodological Maturity. In: Denzin NK and Lincoln YS (eds) The Sage handbook of qualitative research. 5 ed. Los Angeles, Calif: Sage

30. Brinkmann S and Kvale S. *InterViews: learning the craft of qualitative research interviewing.* Thousand Oaks, Calif: Sage. 2015.

31. Grimen H. Profesjon og tillit (Profession and trust). In: Molander A and Terum LI (eds) *Profesjonsstudier (vocational studies)* Oslo: Universitetsforlaget, 2008, pp.197–215.

For peer review only

For peer review only - http://bmjopen.bmj.com/site/about/guidelines.xhtml

32. Skorpen Tarberg A, Landstad BJ, Hole T, Thronæs M, Kvangarsnes M. Nurses' experiences of compassionate care in the palliative pathway. J Clin Nurs. 2020;00:1–9. https://doi.org/10.1111/jocn.15528

33. What Is Health Literacy? https://www.cdc.gov/healthliteracy/learn/index.html (accessed 1 May 2022).

34. Seymour J, Cassel. Palliative care in the USA and England: a critical analysis of meaning and implementation towards a public health approach, *Mortality 2017*, 22:4, 275-290, DOI: 10.1080/13576275.2016.1270262.

For peer review only

Additional file 1 Standards for Reporting Qualitative Research (SRQR)

**Topic**	**Page/line no(s).**
**Title and abstract**	
**Title** - Concise description of the nature and topic of the study Identifying the study as qualitative or indicating the approach (e.g., ethnography, grounded theory) or data collection methods (e.g., interview, focus group) is recommended	1
**Abstract** - Summary of key elements of the study using the abstract format of the intended publication; typically includes background, purpose, methods, results, and conclusions	2
**Introduction**	
**Problem formulation** - Description and significance of the problem/phenomenon studied; review of relevant theory and empirical work; problem statement	4-5
**Purpose or research question** - Purpose of the study and specific objectives or questions	5
**Methods**	
**Qualitative approach and research paradigm** - Qualitative approach (e.g., ethnography, grounded theory, case study, phenomenology, narrative research) and guiding theory if appropriate; identifying the research paradigm (e.g., postpositivist, constructivist/ interpretivist) is also recommended; rationale**	5
**Researcher characteristics and reflexivity** - Researchers' characteristics that may influence the research, including personal attributes, qualifications/experience, relationship with participants, assumptions, and/or presuppositions; potential or actual interaction between researchers' characteristics and the research questions, approach, methods, results, and/or transferability	14-15
**Context** - Setting/site and salient contextual factors; rationale**	5-6
**Sampling strategy** - How and why research participants, documents, or events were selected; criteria for deciding when no further sampling was necessary (e.g., sampling saturation); rationale**	6
**Ethical issues pertaining to human subjects** - Documentation of approval by an appropriate ethics review board and participant consent, or explanation for lack thereof; other confidentiality and data security issues	8
**Data collection methods** - Types of data collected; details of data collection procedures including (as appropriate) start and stop dates of data collection and analysis, iterative process, triangulation of sources/methods, and modification of procedures in response to evolving study findings; rationale**	7
**Data collection instruments and technologies** - Description of instruments (e.g., interview guides, questionnaires) and devices (e.g., audio recorders) used for data collection; if/how the instrument(s) changed over the course of the study	7
**Units of study** - Number and relevant characteristics of participants, documents, or events included in the study; level of participation (could be reported in results)	6
**Data processing** - Methods for processing data prior to and during analysis, including transcription, data entry, data management and security, verification of data integrity, data coding, and anonymization/de-identification of excerpts	7
**Data analysis** - Process by which inferences, themes, etc., were identified and developed, including the researchers involved in data analysis; usually references a specific paradigm or approach; rationale**	8
**Techniques to enhance trustworthiness** - Techniques to enhance trustworthiness and credibility of data analysis (e.g., member checking, audit trail, triangulation); rationale**	14-15
**Results/findings**	
**Synthesis and interpretation** - Main findings (e.g., interpretations, inferences, and themes); might include development of a theory or model, or integration with prior research or theory	8-12
**Links to empirical data** - Evidence (e.g., quotes, field notes, text excerpts, photographs) to substantiate analytic findings	12-15
**Discussion**	
**Integration with prior work, implications, transferability, and contribution(s) to the field -** Short summary of main findings; explanation of how findings and conclusions connect to, support, elaborate on, or challenge conclusions of earlier scholarship; discussion of scope of application/generalizability; identification of unique contribution(s) to scholarship in a discipline or field	14
**Limitations** - Trustworthiness and limitations of findings	14-15
**Other**	
**Conflicts of interest** - Potential sources of influence or perceived influence on study conduct and conclusions; how these were managed	N/A
**Funding** - Sources of funding and other support; role of funders in data collection, interpretation, and reporting	N/A

For peer review only - http://bmjopen.bmj.com/site/about/guidelines.xhtml

*The authors created the SRQR by searching the literature to identify guidelines, reporting standards, and critical appraisal criteria for qualitative research; reviewing the reference lists of retrieved sources; and contacting experts to gain feedback. The SRQR aims to improve the transparency of all aspects of qualitative research by providing clear standards for reporting qualitative research.

**The rationale should briefly discuss the justification for choosing that theory, approach, method, or technique rather than other options available, the assumptions and limitations implicit in those choices, and how those choices influence study conclusions and transferability. As appropriate, the rationale for several items might be discussed together.

Reference:
O'Brien BC, Harris IB, Beckman TJ, Reed DA, Cook DA. **Standards for reporting qualitative research: a synthesis of recommendations.** *Academic Medicine*, Vol. 89, No. 9 / Sept 2014
DOI: 10.1097/ACM.0000000000000388

For peer review only

For peer review only - http://bmjopen.bmj.com/site/about/guidelines.xhtml

BMJ Open

Physicians' narratives of communication with patients and their relatives in different phases of the palliative pathway

Journal:	*BMJ Open*
Manuscript ID	bmjopen-2022-065681.R2
Article Type:	Original research
Date Submitted by the Author:	15-May-2023
Complete List of Authors:	Landstad, Bodil J.; Mid Sweden University Faculty of Human Science, Health Sciences; Östersund Hospital, Unit of Research, Education and Development<br>Kvangarsnes, Marit; Norwegian University of Science and Technology; Møre og Romsdal Hospital Trust
**Primary Subject Heading**:	Palliative care
Secondary Subject Heading:	Communication, General practice / Family practice, Health services research, Palliative care, Qualitative research
Keywords:	Quality in health care < HEALTH SERVICES ADMINISTRATION & MANAGEMENT, Adult palliative care < PALLIATIVE CARE, QUALITATIVE RESEARCH

SCHOLARONE™
Manuscripts

I, the Submitting Author has the right to grant and does grant on behalf of all authors of the Work (as defined in the below author licence), an exclusive licence and/or a non-exclusive licence for contributions from authors who are: i) UK Crown employees; ii) where BMJ has agreed a CC-BY licence shall apply, and/or iii) in accordance with the terms applicable for US Federal Government officers or employees acting as part of their official duties; on a worldwide, perpetual, irrevocable, royalty-free basis to BMJ Publishing Group Ltd ("BMJ") its licensees and where the relevant Journal is co-owned by BMJ to the co-owners of the Journal, to publish the Work in this journal and any other BMJ products and to exploit all rights, as set out in our licence.

The Submitting Author accepts and understands that any supply made under these terms is made by BMJ to the Submitting Author unless you are acting as an employee on behalf of your employer or a postgraduate student of an affiliated institution which is paying any applicable article publishing charge ("APC") for Open Access articles. Where the Submitting Author wishes to make the Work available on an Open Access basis (and intends to pay the relevant APC), the terms of reuse of such Open Access shall be governed by a Creative Commons licence – details of these licences and which Creative Commons licence will apply to this Work are set out in our licence referred to above.

Other than as permitted in any relevant BMJ Author's Self Archiving Policies, I confirm this Work has not been accepted for publication elsewhere, is not being considered for publication elsewhere and does not duplicate material already published. I confirm all authors consent to publication of this Work and authorise the granting of this licence.

For peer review only

Title of the article

Physicians' narratives of communication with patients and their relatives in different phases of the palliative pathway

Authors

1. LANDSTAD Bodil J.*
    a. Faculty of Human Sciences, Mid Sweden University, Sweden
    b. Unit of Research, Education and Development, Östersund Hospital, Östersund, Sweden

    Address: Faculty of Human Sciences, Mid Sweden University, Campus Östersund, 831 25 Östersund, Sweden

    E-mail: bodil.landstad@miun.se

    Phone: +46 730912884

2. KVANGARSNES Marit
    a. Department of Health Sciences, Norwegian University of Science and Technology, Ålesund, Norway
    b. Møre og Romsdal Hospital Trust, Ålesund, Norway

*Corresponding author

Keywords

Physicians, qualitative research, narrative inquiry, communication, palliative care, clinical pathway

Word count

3 996 (excluding title page, abstract, tables, references)

For peer review only - http://bmjopen.bmj.com/site/about/guidelines.xhtml

Abstract

Objectives: To explore physicians' experiences of the communication with patients and their relatives in the different phases of the palliative pathway.

Methods: Purposeful sampling was employed to recruit a total of 13 oncologists and general practitioners who engaged in palliative care. A qualitative study with a narrative approach was conducted. Interviews with physicians working in primary and specialist health care were conducted via Skype Business in the spring of 2020. The interview guide had open-ended questions with each interview lasting between 35 and 60 minutes.

Results: Communication between the physicians, their patients and their relatives was contextual and changed depending on the phase in the palliative pathway. In the first phase, physicians told us that patients and their relatives experienced an emotional shock. Transitioning from the curative to palliative phase was difficult, which emphasised the need for trust through communication. The middle phase, they revealed that communication about the death process became the priority: what was probably going to happen, the family's role in what was going to happen and perhaps, depending on the illness, any medical decisions that needed to be made. It was important for the physicians to communicate information about the palliative pathway while providing the relatives with knowledge that facilitated any decision-making. In the terminal phase physicians employed a compassionate approach, as bereaved family members needed to process their feelings of guilt and grief.

Conclusions: The study gives new insight into communication with patients and their relatives during different phases of the palliative pathway, from the physician's perspective. The findings may help physicians improve the quality of communication with patients and their relatives over these vulnerable pathways. The findings also have practical implications in training contexts. The study reveals ethical dilemmas in physician's communication with patients and their relatives during a palliative pathway.

For peer review only - http://bmjopen.bmj.com/site/about/guidelines.xhtml

Strengths and limitations of this study

- The narrative approach was appropriate when analysing communication in the different phases of the palliative pathway
- The interviews gave a rich and thick data related to different phases in the palliative pathway
- The researchers different professional positions were a strength
- The results are transferable to settings with similar health services

INTRODUCTION

Health care provider's communication with patients and their relatives in palliative care is crucial.[1] A study from New Zealand found the emotional shock and grief that comes with learning of a terminal illness makes it very difficult to hear and process the given health information.[2] Empathy, both as a desirable virtue and as a communication process, has been highlighted in earlier research. Knowing and understanding the patient's perspective can assist clinicians in supporting and guiding the patient through their cancer trajectory.[3]

Communication deficits cause distress for patients and their relatives.[4, 5] The majority of formal complaints in health care are related to communication.[4, 6, 7] Palliative care with patients who have limited health literacy poses particular demands on communication.[8] A Swedish study researching experiences in end-of-life communication showed that there existed a communication barrier when the physicians were uncertain about prognoses, which possibly hindered treatment decisions.[9] The complex nature of the transition from curative to palliative treatment can instill uncertainty and diminish the potential for good dialogues.[9]

For peer review only - http://bmjopen.bmj.com/site/about/guidelines.xhtml

In this study, we used the definition for palliative care provided by the World Health Organization (WHO). Palliative care is an approach that improves the quality of life of patients and their families. Although palliative care is seen as an ethical responsibility, global estimates show that only 14% of patients in need actually receive it.[1] It has been shown to reduce suffering through the early identification, correct assessment and treatment of pain and other problems, whether physical, psychosocial or spiritual'.[1] Palliative care is thus an interdisciplinary approach in which physicians play an important role. As such, this study focused on communication with patients and their relatives by soliciting narratives of communication from the physician's perspective, as this is a highly important component for ensuring quality care.[10] In this context, communication refers to the interpersonal activity of expressing ideas and feelings or providing information; as derived from Latin, communication also refers to 'something you have in common'.[11]

Time and space create the contextual conditions for communication,[12] in which case the physical framework also serves as an important basis. This investigation focused on the type of communication that occurs between physicians, their patients and the patient's relatives in hospitals, home environments, or municipal health service institutions. It is an asymmetrical form of communication with a clear purpose. Here, the patient is in a vulnerable position due to both their dependence on the physician's competence and their access to treatment.[12] As a result of this vulnerability, any such communication is linked to the physician's professional role and position within the specific system, making symmetrical communication undesirable and unprofessional. The relative power held by the physician in this role also entails the risk that patients and their relatives will experience dependency and shame.[13] Research has shown that many patients experience shame, guilt and shortcoming in the communication with the physicians in the treatment process.[13]

Research has shown that in acute and critical situations, the need for shared decision-making is reduced.[14, 15] Trust, therefore, is a central concept in communication between physicians, the patient

For peer review only

and their relatives during the palliative pathway. Physicians have both theoretical and practical knowledge whereas patients and relatives usually have neither; therefore, patients are often required to bestow their trust to physicians for decisions on treatment. The patients presuppose that the physician will act in their best interest. This kind of trust-based relationship is, from the physician's side, ultimately necessary to create the needed space to fully utilize their professional competence.[16]

Sympathy, empathy, and compassion are all important for patients at the end stage of life, but research has shown that patients place the highest value on compassion.[17] A systematic review of 31 studies aimed at communication between healthcare professionals and the relatives of patients approaching the end-of-life stage identified seven relevant themes including highlighting deterioration, involvement in decision-making, post-decision interactional work, tailoring, honesty and clarity, specific techniques for information delivery, and the roles of different healthcare professionals in communication.[18] Patient-centered care and compassion is paramount for patients and relatives during communication in palliative care.[19, 20, 21, 22]

Understanding the patient's level of medical knowledge and understanding, i.e., their health literacy, as well as any other factors that could impact the patient's ability to understand their situation is critical for the physician to accurately relay information. An example of the consequences of not tailoring information to ensure that patients understand was shown in a study of cultural knowledge and understanding in the medical field, which found that a lack of cultural understanding among health professionals led to late access or the avoidance of palliative care among indigenous peoples.[2]

The objective of the study is to explore physicians' communication with patients and their relatives in the different phases of the palliative pathways: the early, middle and, terminal/bereavement phases. We defined these phases as the initial days following the diagnosis of an incurable disease (the early

For peer review only - http://bmjopen.bmj.com/site/about/guidelines.xhtml

phase), the time between the early phase and the last weeks, or days leading up to death (the middle phase), and the last weeks before death and the time after death (terminal/bereavement phase).[23] We therefore posed specific questions in the interview guide to assess the use of communication in the different phases. There is a lack of knowledge about communication in different phases of palliative care from the physician's perspective.

METHODS

The aim of the study was to explore physicians' experiences of the communication with patients and their relatives in the different phases of the palliative pathway. The study has the following research question: How do physicians experience communication with patients and relatives in different phases of the palliative pathway? We employed a narrative approach and subsequent analysis, which entails different theoretical directions that capture personal and human dimensions of experience over time by considering the relationship between individual experiences and cultural contexts.[24] Here, the narrative approach concerned how physicians perceived communication in the early, middle, and terminal/bereavement phases of palliative pathway based on interview data.[25, 26] Narrative analysis emphasizes the physicians voice in different phases of the palliative pathway.[26] The study was compiled with the consolidated criteria for reporting qualitative research (COREQ).[27]

For peer review only

Study population and recruitment

The physicians were recruited by purposive sampling aimed at capturing the complexity of the palliative pathway.[25, 28] The inclusion criteria were physicians with experience from all three phases of care defined in this study.

A contact person recruited a total of 13 physicians working in palliative care, using a direct approach for recruitment through cold calling or face-to-face contact. To ensure diversity and robustness, we recruited males and females of various age groups, experience levels, and medical backgrounds.

For peer review only - http://bmjopen.bmj.com/site/about/guidelines.xhtml

Data collection

We developed an interview guide (Table 1) with open-ended questions based on the study's aim and previous research.[29] This facilitated the solicitation of personal experiences related to communication through the progression of palliative pathway.[30] More specifically, the questions were related to how physicians perceived communication during the different phases of the palliative pathway.

Table 1. Interview guide.

	**Questions**
1	How did you experienced being a physician in palliative care?
2	What is important in communication with patients and relatives in the early phase of the illness?
3	Can you tell about your communication in the middle/terminal phase of the pathway?
4	How do you interact with patients and relatives?
5	What is of importance in communication in the bereavement phase?
6	Is there anything else you want to share with us?

Individual narrative interviews were conducted via Skype Business between April and May 2020. Each interview lasted between 35 and 60 minutes. The interviewer was a researcher as well as cancer nurse, trained to conduct interviews.[27] Saturation was reached upon observing that the data gathered through subsequent interviews yielded redundant information.[25] After the 13 interviews, the data were relating to the different phases of the palliative pathway.

For peer review only - http://bmjopen.bmj.com/site/about/guidelines.xhtml

Patient and public involvement

None

Data analysis

All interviews were audio-recorded and transcribed verbatim. These transcripts were then read to gain an overview of the data.[29] The result section contains stories from 13 physicians that form one narrative. A narrative inquiry research method was chosen as it considers lived experiences and allows for the events that have been of most significance to be brought forth in the stories participants tell. The interviewed physicians had rich and thick experiences from all phases of the palliative pathway. In narrative research the researchers are co-creators in shaping the narrative. A narrative is formed by the way we give meaning to our experiences. This means how we understand our own or others' actions as well as the way we organise the events in our minds. How we connect and perceive the consequences of actions over time is of importance for the shaping of the narrative. Meaning making of our experiences can be linked to the past, the present, and the future.[29]

During a subsequent content analysis, we searched for a plot based on patterns in the narratives. Specifically, this plot described communication during the early, middle, and terminal phases of the palliative pathway.[25, 26]

Ethical consideration

All participants were informed about the study's purpose and procedures, in both oral and written formats. Participation was voluntary and they could withdraw at any time. Further, they were informed that any obtained data would be handled confidentially. The physicians provided informed written consent.

For peer review only - http://bmjopen.bmj.com/site/about/guidelines.xhtml

RESULTS

Oncologists and general practitioners (GPs) who worked in hospitals or primary care settings provided information about how they supported patients and their relatives during different phases of the palliative pathway (see Table 2).

Table 2. Demographic data on the study participants.

	**Participants** (N=13)
**Gender**	
Men	10
Women	3
**Age**	
41-50	7
51-60	4
61-70	2
**Place of assignment***	
Hospital	7
Primary care	7
Private clinic	1
**Education****	
General practitioner	5
Oncologist	5
Physician	5
**Years of experience**	
10-15	2
16-20	5
21-25	-

For peer review only

26-30	4
>30	2

*Some physicians reported more than one workplace.

**Some physicians worked as both oncologists and general practitioners.

We derived the following three themes related to communication in the 1) early, 2) middle, and 3) terminal/bereavement phases, respectively: 1) trusting communication, 2) communication about the death process, and 3) communication about processing relatives' guilt and grief. In Table 3 we present the analysis process for all three themes. The themes illustrate what the physicians considered as significant in the professional communication with patients and relatives in the different phases of the palliative pathway.

Table 3. The analysis process for the three themes.

**Quotation**	**Subtheme**	**Main theme**
Relationship and communication are more important than the patient's choice of treatment	Paternalistic communication	**Trusting communication**
We should take the challenge of talking to patients and relatives together, because they need to hear what the other person is saying - they need to hear together what we are saying	Joint communication	

For peer review only - http://bmjopen.bmj.com/site/about/guidelines.xhtml

Any conveyed information must be adapted to their specific needs	Adapted communication	
We should take the challenge of talking to patients and relatives together, because they need to hear what the other person is saying - they need to hear together what we are saying.	Common understanding	**Communication about the death process**
If we're going to come in and directly inform about prognosis and ask what they want, how they want to die, then I think it's scary - it's too brutal.	Compassion	
We have to accept [situations in which] we do not achieve our treatment goals or a 'good death', which is a value for us. Ain't no cure for death.	Respect for the choices of patients and their relatives	
… it would have been beneficial with another day to	To talk through the situation with the family	

For peer review only - http://bmjopen.bmj.com/site/about/guidelines.xhtml

talk through the situation, or to bring in the family.		
There is not much that needs to be done, a review of the medical history and what actually happened.	Listening to the families experiencing bereavement	**Communication about processing relatives' guilt and grief**
It was important to provide the relatives with stories they could accept and carry with them when going forward.	To give relatives stories to live with	

1. Trusting communication

The physicians described an asymmetrical relationship in which they were the experts. One said the following:

'Relationship and communication are more important than the patient's choice of treatment'.

The narratives revealed that the transition from curative to palliative care as difficult. In addition to the disease-related ailments, patients and their relatives underwent an internalised process of acceptance which often entailed feelings of shock, especially for patients who now realized their limited lifespan. The transition was also often the beginning of a mental process leading to cognition and acceptance of death. Patients had to avail the resources, networks, and coping strategies they built up over the course of their lifetime. Time was marked by loss, and patients learned not expect improvements.

For peer review only

For peer review only - http://bmjopen.bmj.com/site/about/guidelines.xhtml

Physicians expressed they wanted to talk with patients and their relatives together. Here, the goal was to help patients and their relatives develop a common understanding of their situation and the palliative care pathway. One participant said the following:

'We should take the challenge of talking to patients and relatives together, because they need to hear what the other person is saying - they need to hear together what we are saying'.

The physicians experienced that patients and relatives did not have enough knowledge to make decisions about medical treatment choices. The physicians emphasized that patients and relatives in such situations needed experienced physicians to guide them in the course of treatment. This was a way of protecting the patients and their relatives from difficult choices.

This presumed responsibility causes stress for the physicians, who were concerned with being responsibility for the recommended choice of treatment. Being experts in this context, physicians are therefore expected to assess the consequences of the available choices to the patient's specific situation and relay that to the patient. As patients and their relatives often experience crisis during this time, the physicians highlighted that "any conveyed information must be adapted to their [the patient's] specific needs". Practically, the patients must be informed about the disease, the expected course of the disease, and the nature of the treatment during each phase of the pathway. The physicians said it was important to provide this information, by which giving security to patients and their relatives while laying a foundation for appropriate collaboration.

2. Communication about the death process

The physicians said that patients and their relatives continually needed information, but that recognition was a gradual process. One said the following:

For peer review only - http://bmjopen.bmj.com/site/about/guidelines.xhtml

‘We have to take the challenge of asking the difficult questions, and we have to choose the right time to do it. I do not think everyone in the early phases has a realisation that allows us to ask how they want to die’.

‘If we're going to come in and directly inform about prognosis and ask what they want, how they want to die, then I think it's scary – it’s too brutal’.

The physicians conveyed that many patients had ambivalent relationships with death; at times, they would accept their situation, but only a day later express their intent to survive. However, they also said that it was normal and totally acceptable for the patients to have this dual mindset.

Timing is always of essence, especially when communicating with patients, whether to inform or ask questions. Finding the right time to provide patients with information on their disease’s development is very important and that means being cognizant of their patient’s mental status and realizing when the patient is ready to receive this information. As such, the physicians expressed that they spent substantial amounts of time to get an overview of health literacy among patients and their relatives. In their experiences, there were various prerequisites for adapting to situations and processing the nature of disease development at the individual level.

Patient-centered care was also a recurring theme. Here, the physicians reflected on the importance of avoiding situations in which their professional values, e.g., expectations of mastery and a ‘good death’, prevented them from handling difficult situations and enduring ethical dilemmas. In particular, they expressed the need to respect each family’s choices. One physician said the following:

For peer review only - http://bmjopen.bmj.com/site/about/guidelines.xhtml

‘We have to accept the situations in which we do not achieve our treatment goals or a “good death,” which is a value for us. Ain’t no cure for death’.

3. Communication about processing relatives’ guilt and grief

In the terminal/bereavement phase, the physicians said the conflict between efficiency and quality constituted an ethical dilemma in which the demands for efficiency were not always in accordance with the patient’s need for time during the process. One example, noted by a physician, was determining how long a patient should stay at the hospital, saying the following:

‘… it would have been beneficial with another day to talk through the situation, or to bring in the family’.

This recurrent ethical challenge was experienced often by physicians working in hospitals. The terminal phase entails the need for greater involvement of the relatives of patients as it often gives meaning to them during the death process. Having some of the responsibility in caring for their loved ones provided time and space for saying goodbye and experiencing community. There is an importance in listening to the needs of patients and their relatives all the while helping them through the process of acceptance. In this context, it was often beneficial for the physicians to withdraw from the process some, as the patients and their relatives were the true focus. When the process gets to certain point, the focus becomes helping the patients and family come to terms with the situation, as medically there is nothing more that can be done, noted in the following quote:

‘There is not much that needs to be done, a review of the medical history and what actually happened’.

For peer review only - http://bmjopen.bmj.com/site/about/guidelines.xhtml

The process of acceptance for the family is challenging and many relatives often carry guilt moving forward, so 'it was important to provide the relatives with stories they could accept and carry with them when going forward', to help them come to terms with the death. This guilt stems from many areas including that the disease was not detected early enough, or that they had not contributed enough. During bereavement, conversations were thus focused on summarising what occurred over the palliative pathway; the physicians said this helped the relatives end conversations with 'relaxed shoulders'. The atmosphere was often positive, with relatives expressing gratitude and relief. The physicians felt it was important for relatives to talk about any emotional strain they had experienced during the palliative care process. The mourning process was also more conducive in cases where the relatives had contact with physicians early in the process, and thus held continuous dialogues.

DISCUSSION

The narratives showed various experiences with professional communication during different phases of the palliative pathway. In the early phase, the physicians conveyed that, as the patients and their relatives were in crises mode, trustful communication was crucial. Here, they saw it as important to provide relevant information while working to develop a common understanding. In the middle phase, the need for information was strong with the patients and their families having many questions. Compassion, respect and a common understanding are considered significant in this dialog and more importantly for the physicians was the timing of the information given. Following this phase into the final and terminal phase, the bereaved relatives needed experiences, interactions and memories that could help them through the acceptance process. As such, conversations during bereavement helped the mourning process and assuaged feelings of guilt and grief.

The transitioning from the curative to palliative phase is difficult for all involved making the dialog between the physicians and the patient and their families even more important. In palliative care,

For peer review only - http://bmjopen.bmj.com/site/about/guidelines.xhtml

the goal of the communication is to develop a common understanding of the coming situation that will occur. This dialog is a way to create trust, which is a central component.[31]

Previous studies have emphasised that patients and their relatives depend on expert knowledge from physicians making the doctor-patient relationship asymmetrical due to professional roles and other factors.[12, 16] Research has shown that in acute and critical situations, the need for shared decision-making is reduced.[14, 15] Physicians must therefore consider issues and needs from the patient's perspective, as they are entrusted with the care of the patient 'in good faith'. In this study, the physicians said their patients conferred trust when allowing them to decide the best course of treatment over the palliative pathway. In other words, patients assume that physicians will act in their best interests. In turn, this creates the space that physicians need to fully use their professional competencies. While this forms a basis for exercising benign power, it is also important to remember that such trust places the patient in a position of vulnerability to negative forms of power.[16] On the other hand, uncertainty about the prognosis may hinder communication between physicians when attempting to provide information about palliative treatment,[9] which likely imposes a variety of other communicative difficulties. In sum, clear communication is necessary for developing trust during vulnerable health situations.[9, 17, 16]

We found that timing was important when providing information about disease prognosis. The physicians said they needed to ask difficult questions at the right time, which required an understanding of individual characteristics, including various levels of health literacy among patients and their relatives. The physicians said they did not always achieve their intention of facilitating 'a good death' for the patient, as it was considered important to respect and comply with the wishes of patients and their families. Compassion is widely considered an essential element in palliative care.[20, 21, 32] A previous study reported that compassion and empathy were preferred over sympathy from

For peer review only - http://bmjopen.bmj.com/site/about/guidelines.xhtml

the perspective of the patient on palliative care.[17] Compassion, in this case, being the most preferred, as it benefited communication addressing their suffering and needs through relational understanding and action.[17] This definition also emphasises action as an important aspect of compassion, which reflects the practices implemented by this study's physicians, who prioritised the preferences of patients and their relatives over their own professional values during care. Other studies have also highlighted the importance of tailored, clear, and patient-centered communication in facilitating death.[18, 22] Further, cultural competence is highly important when adapting information to the various levels of health literacy among patients and their relatives.[18] Personal health literacy is defined as the ability to find, understand, and use information and services to make health-related decisions and actions for themselves and others.[33] In this study, physicians said it was important to map the health literacy to understand the level of comprehension of the patient and their family members. In doing so, the physician was better adept to change how they spoke to those involved ensuring their message was received and understood as intended.

Following the death of the patient, physicians stated that continued communication during bereavement was important. Relatives needed stories they could relate to within their own lives. In the mourning process, it was helpful when physicians had been involved from early on and were thus able to hold continual dialogues with the relatives. However, research has shown that physicians come into the picture at a later stage in the process and many relatives are not given the opportunity to have these conversations.[32] In sum, both the current and previous findings emphasise that the continuous dialog during bereavement are important aspects of the palliative care process.

For peer review only - http://bmjopen.bmj.com/site/about/guidelines.xhtml

Conclusion

The study gives new insight into physician's experiences of communication in different phases of the palliative pathway. Trustful communication with patients and their relatives were crucial in the first phase. In the middle phase communication about the death process is important and, respect for the choices of patients and their relatives were important. A common understanding with the patient and their relatives of the treatment was paramount. In the bereavement phase, talking through the situation with the relatives and giving them stories to live with was of great significance for the physicians.

A conclusion is that the physicians may experience an ethical dilemma in communication with patients and their relatives about the efficiency and quality in the treatment. Conflicts might arise between the physicians and the patient and relatives in, for example, treatment choices. Taking a paternalistic attitude to protect the patient from harm can be morally complicated. In health care treatment the physicians have superior knowledge and insights and thus, in an authoritative position to determine the patient's best interests.[34] A conflict between beneficence and respect for the patient and the relative's autonomy to participate in treatment choices may arise. Physicians should be prepared for such ethical dilemmas. Ethical dilemmas in palliative care should be an important topic of discussion in the community of practices of physicians as well as in medical education.

Strengths and limitations

This study is limited to the experiences of the physicians. No patient and public involvement is included in the study. The qualitative interview approach provided rich and complex data, as we employed an explicitly theoretical framework, thus enhancing trustworthiness.[12, 16, 30] Further, direct quotations underpinned the main findings.[27] The researcher's different professional backgrounds, being RN and social scientists, aided in understand the complexity of the obtained narratives. Such intersubjective understanding also improved the study's authenticity.[26] Member checking was done

For peer review only - http://bmjopen.bmj.com/site/about/guidelines.xhtml

during the interview process by an experienced oncologist. The study was solely conducted in the Norwegian context. Nevertheless, research suggests that our findings may be relevant in other cultural contexts such as New Zealand, Europe and USA.[2, 35]

Implications of results for practice and further research

In this study, the narrative approach revealed new insights into how physicians communicate with patients and their relatives during different phases of palliative care. In practice, these findings should help improve the overall quality of communication. A close collaboration with relatives in the terminal phase is important for achieving a 'good death'. Additional studies should continue to investigate the issue with cultural dimension of communication in palliative care. It is also of interest to study communication within the team working between health professionals in palliative care. The digital age creates new ways of communicating and it would be interesting to explore the possibilities of this interaction in palliative care.

Conflict of interests

The authors report no conflicts of interests.

Funding

No funding to report.

Acknowledgment

We express our gratitude to all participating physicians who shared their experiences about communication over the palliative pathway.

For peer review only - http://bmjopen.bmj.com/site/about/guidelines.xhtml

Contributorship statement

Conceptualization, BJL and MK; methodology, BJL and MK; validation, BJL and MK; formal analysis, BJL and MK; investigation, BJL and MK; writing - original draft preparation, BJL and MK; writing - review and editing, BJL and MK. Both authors have read and agreed to the published version of the manuscript.

Ethics statement

According to the Regional Committee on Medical and Health Research Ethics, this study did not require approval (REK 78067). However, we did receive approval from the Data Protection Officer associated with the Møre og Romsdal Hospital Trust (HMR 2020/397-2) and the Norwegian Centre for Research Data (NSD 131948).

Data Sharing Statement

The interviews are transcribed verbatim in specific Norwegian dialects and it is therefore not appropriate to share the data with other researchers. The dialects are very different from the two Norwegian official languages *Bokmål* and *Nynorsk* and therefore not appropriate to share.

REFERENCES

For peer review only - http://bmjopen.bmj.com/site/about/guidelines.xhtml

1. World Health Organization (WHO). *Palliative care*. https://www.who.int/news-room/fact-sheets/detail/palliative-care (accessed 1 May 2022).

2. Kidd J, Black S, Blundell R, et al. Cultural health literacy: the experiences of Māori in palliative care. *Glob Health Promot* 2018; 25(4): 15–23.

3. van Vliet LM & Back AL. The different faces of empathy in cancer care: From a desired virtue to an evidence-based communication process. *Cancer* 2021;127:4137-4139 https://doi.org/10.1002/cncr.33833

4. van Vliet LM, Epstein AS. The current state of the art and science of patient-clinician communication in progressive disease: Patients' need to know and need to feel known. J Clin Oncol 2014;32:3474-8 https://pubmed.ncbi.nlm.nih.gov/25267758/

5. Morita T, Akechi T, Ikenaga T, et al: Communication about the ending of anticancer treatment and transition to palliative care, *Ann Oncol* 15: 1551– 1557, 2004.

6. Pincock S: Poor communication lies at heart of NHS complaints, says ombudsman, *BMJ*, 2004, 328: 10.

7. Tamblyn R, Abrahamowicz M, Dauphinee D, et al: Physician scores on a national clinical skills examination as predictors of complaints to medical regulatory authorities, *JAMA*, 2007, 298: 993–1001.

For peer review only - http://bmjopen.bmj.com/site/about/guidelines.xhtml

8. Roodbeen R, Vreke A, Boland G, Rademakers J, van den Muijsenbergh M, Noordman J, et al. (2020) Communication and shared decision-making with patients with limited health literacy; helpful strategies, barriers and suggestions for improvement reported by hospital-based palliative care providers. *PLoS ONE* 15(6): e0234926. https://doi.org/10.1371/journal.pone.0234926

9. Udo C, Lövgren M, Lundquist G, et al. Palliative care physicians' experiences of end-of-life communication: a focus group study. *Eur J Cancer Care* 2018; 27: e12728.

10. Cain C, Surbone A, Elk R, et al. Culture and palliative care: preferences, communication, meaning, and mutual decision making. *J Pain Symptom Manage* 2018; 55: 1408e1419.

11. Oxford Learner's Dictionaries. Communication. https://www.oxfordlearnersdictionaries.com/definition/english/communication?q=communication (accessed 1 May 2022).

12. Madssen K-A. Språk og kommunikasjon: Myndiggjering eller umyndiggjering? Kap. 9. In: Ekeland TJ and Heggen K (eds) *Meistring og myndiggjering. Reform eller retorikk?* Oslo: Gyldendal Akademisk, 2007.

13. Guldbrandsen P, Fugellie P, Stang G, et al. *Skam i det medisinske rom.* Oslo: Gyldendal akademisk. 2006.

14. Bårdsgjerde EK, Kvangarsnes M, Landstad B, Nylenna M, Hole T. Patients' narratives of their patient participation in the myocardial infarction pathway. *J Adv Nurs*. 2019;75:1063-1073. https://doi.org/10.1111/jan.13931

For peer review only

15. Thompson, A. G. H. (2007). The meaning of patient involvement and participation in health care consultations: A taxonomy. *Social Science & Medicine*, 64(6), 1297–1310. https://doi.org/10.1016/j.socscimed. 2006.11.002

16. Grimen H. *Hva er tillit? (What is trust?)* Oslo: Univeristetsforlaget, 2009.

17. Sinclair S, Beamer K, Hack TF, et al. Sympathy, empathy, and compassion: a grounded theory study of palliative care patients' understandings, experiences, and preferences. *Palliat Med* 2017; 31(5): 437–447.

18. Anderson R, Bloch S, Armstrong M, et al. Communication between healthcare professionals and relatives of patients approaching the end-of-life: a systematic review of qualitative evidence. *Palliat Med* 2019; 33(8): 926–941.

19. Lavoie M, Blondeau D and Martineau I. The integration of a person-centered approach in palliative care. *Palliat Support Care* 2013; 11: 453–464.

20. Crawford P, Brown B, Kvangarsnes M, et al. The design of compassionate care. *J Clin Nurs* 2014; 23(23-24): 3589-99.

21. Mills J, Wand T and Fraser JA. Palliative care professionals' care and compassion for self and others: a narrative review. *Int J Palliat Nurs* 2017; 23(5): 219-229.

22. Schellinger SE, Anderson EW, Frazer, MS, et al. Patient self-defined goals: essentials of person-centered care for serious illness. *Am J Hosp Palliat Med* 2018; 35(1): 159-165.

For peer review only - http://bmjopen.bmj.com/site/about/guidelines.xhtml

23. Tarberg Skorpen A, Kvangarsnes M, Hole T, et al. Silent voices – family caregivers' narratives of palliative pathways. *Nurs Open* 2019; 00: 1–9.

24. Clandinin DJ and Connelly FM. *Narrative inquiry: experience and story in qualitative research.* San Francisco: Jossey-Bass, 2000.

25. Patton MQ. *Qualitative research & evaluation methods: integrating theory and practice.* Los Angeles, California: Sage, 2015.

26. Holloway I and Freshwater H. *Narrative research in nursing.* Oxford, UK: Blackwell, 2007.

27. Tong, A., Sainsbury, P., & Craig, J. (2007). Consolidated criteria for reporting qualitative research (COREQ): a 32-item checklist for interviews and focus groups. *International journal for quality in health care*, 19(6), 349-357. doi:10.1093/intqhc/mzm042

28. Morse JM and Richards L. *Read me first for a user's guide to qualitative methods*. Thousand Oaks, California: Sage, 2002.

29. Chase SE (2018) Narrative Inquiry Toward Theoretical and Methodological Maturity. In: Denzin NK and Lincoln YS (eds) *The Sage handbook of qualitative research*. 5 ed. Los Angeles, Calif: Sage

30. Brinkmann S and Kvale S. *InterViews: learning the craft of qualitative research interviewing.* Thousand Oaks, Calif: Sage. 2015.

31. Grimen H. *Profesjon og tillit* (Profession and trust). In: Molander A and Terum LI (eds) *Profesjonsstudier (vocational studies)* Oslo: Universitetsforlaget, 2008, pp.197–215.

For peer review only

For peer review only - http://bmjopen.bmj.com/site/about/guidelines.xhtml

32. Skorpen Tarberg A, Landstad BJ, Hole T, Thronæs M, Kvangarsnes M. Nurses' experiences of compassionate care in the palliative pathway. *J Clin Nurs*. 2020;00:1–9. https://doi.org/10.1111/jocn.15528

33. What Is Health Literacy? https://www.cdc.gov/healthliteracy/learn/index.html (accessed 1 May 2022).

34. Beauchamp, T.L., Childress, J.F. (2019) *Principles of Biomedical Ethics* (8th ed). New York: Oxford University Press.

35. Seymour J, Cassel. Palliative care in the USA and England: a critical analysis of meaning and implementation towards a public health approach, *Mortality 2017*, 22:4, 275-290, DOI: 10.1080/13576275.2016.1270262.

For peer review only

For peer review only - http://bmjopen.bmj.com/site/about/guidelines.xhtml

Additional file 1 Standards for Reporting Qualitative Research (SRQR)

Topic	Page/line no(s).
**Title and abstract**	
**Title** - Concise description of the nature and topic of the study Identifying the study as qualitative or indicating the approach (e.g., ethnography, grounded theory) or data collection methods (e.g., interview, focus group) is recommended	1
**Abstract** - Summary of key elements of the study using the abstract format of the intended publication; typically includes background, purpose, methods, results, and conclusions	2
**Introduction**	
**Problem formulation** - Description and significance of the problem/phenomenon studied; review of relevant theory and empirical work; problem statement	4-5
**Purpose or research question** - Purpose of the study and specific objectives or questions	5
**Methods**	
**Qualitative approach and research paradigm** - Qualitative approach (e.g., ethnography, grounded theory, case study, phenomenology, narrative research) and guiding theory if appropriate; identifying the research paradigm (e.g., postpositivist, constructivist/ interpretivist) is also recommended; rationale**	5
**Researcher characteristics and reflexivity** - Researchers' characteristics that may influence the research, including personal attributes, qualifications/experience, relationship with participants, assumptions, and/or presuppositions; potential or actual interaction between researchers' characteristics and the research questions, approach, methods, results, and/or transferability	14-15
**Context** - Setting/site and salient contextual factors; rationale**	5-6
**Sampling strategy** - How and why research participants, documents, or events were selected; criteria for deciding when no further sampling was necessary (e.g., sampling saturation); rationale**	6
**Ethical issues pertaining to human subjects** - Documentation of approval by an appropriate ethics review board and participant consent, or explanation for lack thereof; other confidentiality and data security issues	8
**Data collection methods** - Types of data collected; details of data collection procedures including (as appropriate) start and stop dates of data collection and analysis, iterative process, triangulation of sources/methods, and modification of procedures in response to evolving study findings; rationale**	7
**Data collection instruments and technologies** - Description of instruments (e.g., interview guides, questionnaires) and devices (e.g., audio recorders) used for data collection; if/how the instrument(s) changed over the course of the study	7
**Units of study** - Number and relevant characteristics of participants, documents, or events included in the study; level of participation (could be reported in results)	6
**Data processing** - Methods for processing data prior to and during analysis, including transcription, data entry, data management and security, verification of data integrity, data coding, and anonymization/de-identification of excerpts	7
**Data analysis** - Process by which inferences, themes, etc., were identified and developed, including the researchers involved in data analysis; usually references a specific paradigm or approach; rationale**	8
**Techniques to enhance trustworthiness** - Techniques to enhance trustworthiness and credibility of data analysis (e.g., member checking, audit trail, triangulation); rationale**	14-15
**Results/findings**	
**Synthesis and interpretation** - Main findings (e.g., interpretations, inferences, and themes); might include development of a theory or model, or integration with prior research or theory	8-12
**Links to empirical data** - Evidence (e.g., quotes, field notes, text excerpts, photographs) to substantiate analytic findings	12-15
**Discussion**	
**Integration with prior work, implications, transferability, and contribution(s) to the field -** Short summary of main findings; explanation of how findings and conclusions connect to, support, elaborate on, or challenge conclusions of earlier scholarship; discussion of scope of application/generalizability; identification of unique contribution(s) to scholarship in a discipline or field	14
**Limitations** - Trustworthiness and limitations of findings	14-15
**Other**	
**Conflicts of interest** - Potential sources of influence or perceived influence on study conduct and conclusions; how these were managed	N/A
**Funding** - Sources of funding and other support; role of funders in data collection, interpretation, and reporting	N/A

For peer review only - http://bmjopen.bmj.com/site/about/guidelines.xhtml

*The authors created the SRQR by searching the literature to identify guidelines, reporting standards, and critical appraisal criteria for qualitative research; reviewing the reference lists of retrieved sources; and contacting experts to gain feedback. The SRQR aims to improve the transparency of all aspects of qualitative research by providing clear standards for reporting qualitative research.

**The rationale should briefly discuss the justification for choosing that theory, approach, method, or technique rather than other options available, the assumptions and limitations implicit in those choices, and how those choices influence study conclusions and transferability. As appropriate, the rationale for several items might be discussed together.

Reference:

O'Brien BC, Harris IB, Beckman TJ, Reed DA, Cook DA. **Standards for reporting qualitative research: a synthesis of recommendations.** *Academic Medicine*, Vol. 89, No. 9 / Sept 2014
DOI: 10.1097/ACM.0000000000000388

For peer review only
